# Exploring genetic susceptibility to air pollution and its implications for disease risk and precision health: A scoping review

**DOI:** 10.3934/publichealth.2025046

**Published:** 2025-09-11

**Authors:** Hari Krismanuel

**Affiliations:** Faculty of Medicine, Universitas Trisakti, West Jakarta, DKI Jakarta, Indonesia

**Keywords:** air pollution, disease risk, environmental health, genetic susceptibility, personalized medicine, precision health

## Abstract

Air pollution, comprising a complex mixture of gaseous and particulate pollutants, remains a major global health concern that disproportionately affects vulnerable populations. In this scoping review, we aim to systematically investigate the role of genetic susceptibility in health outcomes associated with exposure to air pollution, with a particular emphasis on fine particulate matter (PM_2.5_), particulate matter (PM_10_), nitrogen dioxide (NO_2_), and nitrogen oxides (NO_x_); key pollutants consistently linked to adverse health effects. By exploring the gene-environment interactions underlying air pollution-related conditions, this review offers new insights into how genetic factors may modulate individual responses to air pollutants and their implications for precision health. Analyzing 16 peer-reviewed studies published in the last decade, we highlight genetic markers and pathways involved in regulating oxidative stress, inflammation, and DNA repair, which are thought to influence individual variation in responses to PM_2.5_, PM_10_, NO_2_, and NO_x_. Although none of the included studies entailed multi-omics or machine learning approaches, we identified these tools as promising directions for future research aimed at elucidating mechanistic pathways and informing personalized strategies. These techniques could significantly improve the understanding of gene-environment interactions, and are suggested as emerging methodologies for future studies. However, the scarcity of longitudinal studies and the underrepresentation of diverse populations limit the generalizability of the current findings. Addressing these gaps will be essential for advancing research, improving environmental health equity, and informing policy in the context of air pollution and genetic susceptibility.

**Figure 1.**
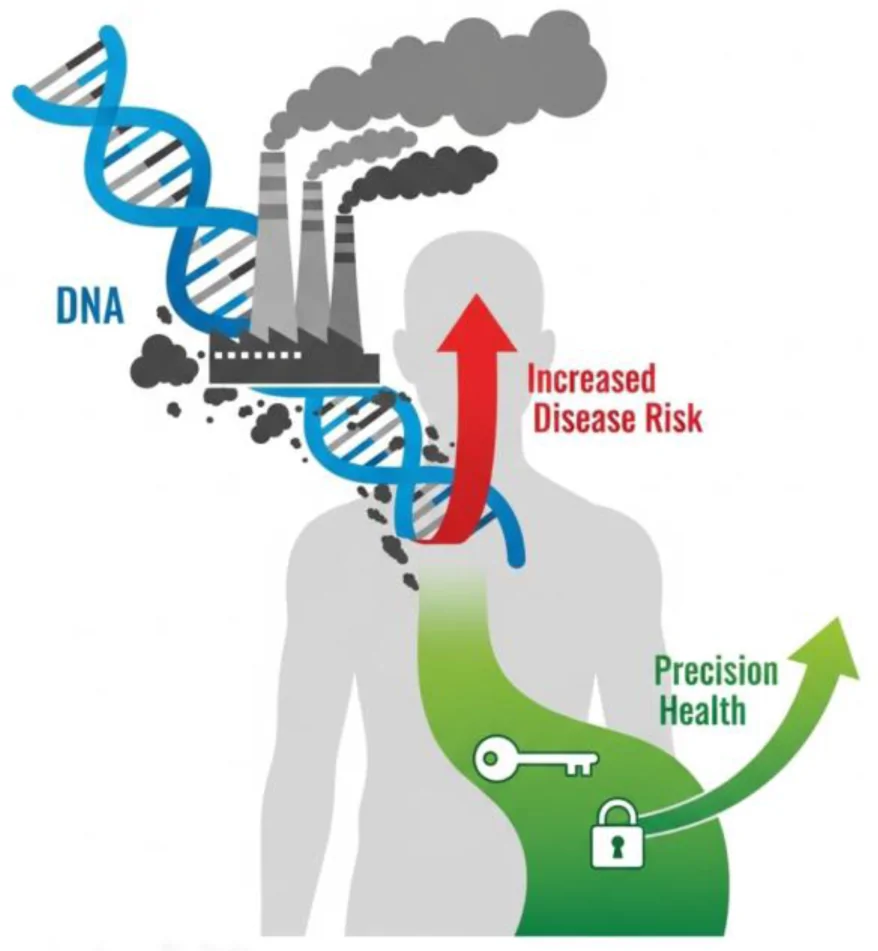
Graphical abstract.

Figure 1 illustrates the conceptual pathway from the interaction of **genetic susceptibility** (DNA helix) and **air pollution exposure** (smokestacks), which leads to an **increased disease risk** in individuals. The green pathway highlights how **precision health** strategies, tailored to an individual's unique genetic and environmental profile, can serve as a targeted solution to mitigate this risk.

## 1. Introduction

Air pollution remains one of the most significant environmental risk factors worldwide, contributing to an estimated 7 million premature deaths annually, according to the World Health Organization [1]–[3]. Among the most harmful pollutants are fine particulate matter (PM_2.5_), coarse particulate matter (PM_10_), nitrogen dioxide (NO_2_), and nitrogen oxides (NO_x_), which are consistently associated with adverse health outcomes [4]–[6].

**PM_2.5_ and PM_10_** refer to airborne particles with aerodynamic diameters ≤2.5 and ≤10 micrometers, respectively. These particles can penetrate deep into the respiratory tract, triggering oxidative stress, inflammation, endothelial dysfunction, and systemic effects beyond the lungs. **NO_2_ and NO_x_**, primarily emitted from vehicle exhaust and industrial processes, contribute to airway inflammation, impaired lung function, and increased cardiovascular risk. Exposure to these pollutants has been linked to the development and exacerbation of chronic diseases such as asthma, chronic obstructive pulmonary disease (COPD), ischemic heart disease, stroke, and neurodegenerative conditions [4]–[6].

Recent fine-scale modeling and exposure assessment studies, such as that of Nisticò et al. (2025), emphasize the importance of high-resolution pollution data in identifying vulnerable populations and guiding local-level interventions. Understanding the complex interplay between environmental exposures and individual susceptibility, particularly at the molecular level, is crucial for developing targeted public health interventions. This necessitates the integration of detailed environmental monitoring data with comprehensive health surveillance and molecular research, including the investigation of genetic factors that may modify an individual's response to air pollution [7].

Genetic susceptibility to air pollution refers to the predisposition of certain individuals to experience heightened adverse health effects due to specific genetic variations. Genes involved in oxidative stress pathways play critical roles in neutralizing reactive oxygen species generated by pollutants like fine particulate matter (PM_2.5_). Understanding these genetic mechanisms is crucial for explaining why some populations exhibit increased vulnerability to air pollution-related diseases [8]–[10].

Air pollution remains a major global health challenge, imposing significant health burdens worldwide. Primary pollutants, such as PM_2.5_, nitrogen dioxide (NO_2_), ozone, and volatile organic compounds (VOCs), are widely acknowledged as key contributors to diseases across multiple systems. However, while environmental exposures are well-documented as primary drivers, genetic variations significantly modulate individual susceptibility, disproportionately affecting vulnerable populations. Despite its importance, the interaction between genetic predisposition and pollutant exposure remains underexplored, leaving critical gaps in our understanding of the mechanisms driving health disparities [11]–[13].

Recent advancements in genetic research have illuminated how genetic variants influence sensitivity to oxidative stress, inflammation, DNA damage, and epigenetic modifications, all of which are implicated in pollution-related diseases. However, significant challenges persist, including inconsistent findings across studies due to methodological differences and the underrepresentation of diverse populations in genetic analyses. Genome-wide association studies (GWAS) have identified promising genetic markers, yet these findings often lack generalizability due to limited population diversity and a lack of comprehensive models that integrate genetic and environmental factors [14],[15].

To address these gaps, emerging methodologies such as multi-omics integration and machine learning are increasingly recognized as powerful tools to uncover complex gene-environment interactions. While these techniques were not employed in the studies included in this review, they hold great promise for future research aimed at identifying mechanistic pathways and advancing precision health strategies [16]–[19].

In this review, we address these gaps by systematically analyzing 16 peer-reviewed studies published over the past decade to provide a detailed synthesis of the interplay between genetic and environmental factors in determining health risks associated with air pollution. By focusing on oxidative stress, inflammation, and epigenetic pathways, we uniquely highlight genetic mechanisms that modulate susceptibility to pollution-related diseases. We also identify critical research gaps, such as the reliance on cross-sectional designs, and propose future directions to improve the robustness and generalizability of findings.

We further aim to outline a novel framework for advancing precision health strategies by integrating genetic insights with emerging methodologies such as multi-omics, machine learning, and longitudinal study designs. By doing so, we seek to inform public health policies aimed at mitigating air pollution-related health risks, particularly in vulnerable populations.

A detailed overview of the included studies, including author, year, location, study design, population and sample size, exposure variables, health outcomes, and age range, is presented in Supplementary Table S1. This table provides a comprehensive summary of the key characteristics of the included studies, enabling comparison and the identification of research gaps.

Despite the growing body of epidemiological research, the underlying biological mechanisms of gene-environment (GxE) interactions remain complex and not fully understood. In addition to epidemiological studies, mechanistic data from in vivo and organoid models also provide crucial insights into the biological pathways underlying GxE interactions. Researchers have demonstrated how such models can elucidate the cellular responses to environmental exposures in genetically predisposed individuals [8],[20],[21], which are discussed further in the Discussion section.

## 2. Materials and methods

### 2.1. Protocol and registration

This scoping review was conducted following the methodological framework proposed by Arksey and O'Malley (2005) [22] and further elaborated by Levac et al. (2010) [23]. Recognizing the importance of transparency and methodological rigor for evidence synthesis, the protocol for this scoping review was retrospectively registered with the Open Science Framework (OSF) on May 22, 2025. The public URL for this registration is https://osf.io/3r8ap/ and its Registration ID is 3r8ap. This protocol is publicly available on the OSF platform [24].

### 2.2. Search strategy

To ensure transparency and credibility, a systematic literature search was conducted across multiple databases, including PubMed, Google Scholar, and ResearchGate to identify relevant studies. The search was limited to articles published in English between January 1, 2015, and December 31, 2024. The following search strategy was used:

- **PubMed:** (“air pollution” [MeSH Terms] OR “air pollution” [Title/Abstract] OR “air pollutants” [Title/Abstract]) AND (“genetic susceptibility” [MeSH Terms] OR “genetic polymorphism” [Title/Abstract] OR “oxidative stress” [MeSH Terms] OR “oxidative stress” [Title/Abstract]) AND (“disease risk” [Title/Abstract] OR “health outcomes” [Title/Abstract]).
- **Google Scholar:** “air pollution” AND (“genetic susceptibility” OR “oxidative stress”) AND (“disease risk” OR “health outcomes”).
- **ResearchGate:** (“air pollution” OR “air pollutants” OR “pencemaran udara”) AND (“genetic susceptibility” OR “genetic predisposition” OR “oxidative stress” OR “stress oksidatif”) AND (“disease risk” OR “health outcomes” OR “dampak kesehatan”).
- **DOAJ**: “air pollution” AND (“genetic susceptibility” OR “oxidative stress”) AND (“disease risk” OR “health outcomes”).

The following filters were applied: Human studies, English language, publication date (2015–2024), study type (including review, meta-analysis, randomized controlled trial, cohort study, case-control study, and cross-sectional study), and peer-reviewed status.

### 2.3. Study selection process

Articles were screened for relevance using a two-step process: (1) Title and abstract screening, followed by (2) full-text review. From this systematic search, 16 peer-reviewed articles were selected based on their relevance to the topic. Data from the selected articles were then systematically extracted. Data extraction prioritized information on genetic markers, their roles in modulating susceptibility, and their associations with health effects induced by air pollution. The data synthesis employed a qualitative approach to integrate findings from these studies, focusing on the influence of genetic factors on susceptibility to air pollution and the interaction between genetic variations and environmental exposures. This enabled the identification of patterns and relationships between genetic variations and health risks associated with air pollution, providing a comprehensive perspective on how genetics influences responses to environmental pollutants [25]–[27].

To ensure the transparency and reproducibility of this review, the study selection process was guided by the PRISMA-ScR (Preferred Reporting Items for Systematic Reviews and Meta-Analyses extension for Scoping Reviews) framework. A PRISMA-ScR flow diagram was used to illustrate the process of study selection, and adherence to PRISMA guidelines was maintained throughout the data extraction and synthesis phases [28]–[30].

**Step 1: Identifying Studies.** Relevant studies were initially identified through a comprehensive search across multiple databases, including PubMed, Web of Science, and Google Scholar. A combination of keywords like “air pollution,” “genetic susceptibility,” “oxidative stress,” and “disease risk” was used to locate pertinent articles. These searches aimed to capture a broad range of studies related to genetic factors and their interactions with environmental exposures.

The search results were carefully reviewed, and studies meeting the predefined inclusion criteria were selected for further assessment. Studies that did not meet the inclusion criteria, were not substantially relevant to the research topic, or contained duplicated references were excluded.

This step ensured the selection of studies that contribute meaningful and relevant insights to the review, avoiding redundancy and maintaining the quality and integrity of the synthesis [28]–[30].

**Step 2: Study Screening.** The next step involved screening the identified studies based on predefined inclusion and exclusion criteria. Two reviewers independently screened the titles and abstracts of the studies retrieved from the initial search. Studies were selected for inclusion if they met the following criteria:

- Focused on genetic susceptibility to air pollution.
- Provided explicit methodologies.
- Offered quantitative or mechanistic insights into genetic-environment interactions.

Studies that were excluded at this stage included those not published in English, non-peer-reviewed articles, conference abstracts, and reviews that did not directly address genetic susceptibility to air pollution. The remaining articles underwent a full-text review to confirm their eligibility before being included in the final analysis [28]–[30].

**Step 3: Data Extraction.** Data were extracted from the selected studies using a standardized extraction form. The extraction process involved collecting detailed information on genetic markers, biomarkers, health outcomes related to air pollution exposure, and other relevant details like study design, sample size, and key findings. The data were then synthesized qualitatively to identify key themes, patterns, and relationships across the studies [28]–[30].

**Step 4: Data Synthesis.** Data synthesis involved integrating findings from the selected studies to draw conclusions about the influence of genetic factors on susceptibility to air pollution. This synthesis aimed to provide a comprehensive understanding of the mechanisms underlying genetic-environment interactions and their implications for disease risk. The integration of findings was guided by thematic analysis and narrative synthesis techniques, emphasizing consistency and comparability across studies [28]–[30].

### 2.4. Inclusion and exclusion criteria

Studies were included in this scoping review if they met the following criteria:

#### 2.4.1. Inclusion criteria

##### 2.4.1.1. Study design

Studies of any design that investigated the association between air pollution exposure (e.g., PM_2.5_, PM_10_, NO_2_, and NO_x_) and health outcomes in relation to genetic susceptibility were included. This encompasses observational studies (cohort, case-control, cross-sectional), interventional studies (e.g., randomized controlled trials, and quasi-experimental studies), and Mendelian Randomization studies. Scoping reviews are particularly suitable for mapping evidence on complex and heterogeneous topics, as outlined by Tricco et al. (2018) [28], Page et al. (2021) [29], and Page and Moher (2017) [30]. The focus was on studies examining various genetic factors influencing susceptibility to air pollution than specific genetic polymorphisms.

##### 2.4.1.2. Population

Human participants of any age, sex, or ethnicity. Studies focusing on specific subpopulations (e.g., children, elderly, and individuals with specific pre-existing conditions) were also included.

##### 2.4.1.3. Exposure

Measurable exposure to PM_2.5_, PM_10_, NO_2_, or NO_x_. Studies must provide quantitative or qualitative data on one or more of these pollutants. Exposure assessment methods should be clearly described (e.g., air quality monitoring data, self-reported exposure, and residential proximity to pollution sources).

##### 2.4.1.4. Health outcomes

Any health outcomes relevant to the research question, including but not limited to respiratory diseases (e.g., asthma, and COPD), cardiovascular diseases, mental health effects, pregnancy complications, and skin conditions. Studies must report specific health outcomes and diagnostic criteria used.

##### 2.4.1.5. Gene-environment interaction (primary and essential criterion)

Studies must present statistical analyses that directly test for a gene-environment interaction (e.g., using interaction terms in regression models, stratified analyses by genotype, interaction meta-regression). Studies reporting only the major effects of air pollution or genetic associations separately were excluded. Studies that mention gene-environment interaction but did not perform formal statistical testing of the interaction were also excluded [8],[31],[32].

#### 2.4.2. Exclusion criteria

Studies were excluded if they met any of the following criteria:

##### 2.4.2.1. Irrelevance to the topic

○ Studies that did not address the health effects of air pollution.

○ Studies that focused exclusively on pollutants other than PM_2.5_ (e.g., only NO_2_ or O_3_).

○ Studies addressing PM_2.5_ along with other pollutants were considered if PM_2.5_-specific information could be extracted.

○ Studies that entailed the environmental impact of air pollution but not human health effects.

○ Studies solely focused on interventions or policies to reduce air pollution without addressing genetic aspects.

##### 2.4.2.2. Lack of genetic focus

○ Purely epidemiological studies that measured only air pollution exposure and health outcomes without considering genetic factors.

○ *In vitro* or *in vivo* toxicological studies that did not investigate genetic variations or gene polymorphisms.

##### 2.4.2.3. Inappropriate publication type

○ Opinions, editorials, letters to the editor, and conference abstracts (unless the abstracts contained significant information not available in a full-text publication).

○ Books and book chapters (unless they contained relevant systematic reviews or meta-analyses).

○ Government or non-governmental organization reports (unless they contained significant data or analyses not available in peer-reviewed publications).

##### 2.4.2.4. Language and accessibility

○ Studies not published in languages accessible to the review team (e.g., English and Indonesian).

○ Studies for which full-text access could not be obtained after reasonable search efforts (e.g., through library databases or direct requests to authors).

##### 2.4.2.5. Duplication

○ Studies published more than once (in which case, the most complete and recent version was included).

##### 2.4.2.6. Methodological concerns (with specific consideration for scoping reviews)

○ While scoping reviews generally do not assess the methodological quality of studies as rigorously as systematic reviews, studies with substantial methodological flaws (e.g., severely flawed study design or erroneous data analysis) could be excluded. This criterion was applied cautiously and transparently [33]–[34].

### 2.5. Data extraction and synthesis

Data from included studies were extracted using a standardized data extraction form. The following information was extracted: Study characteristics (e.g., author, year, study design, and population), exposure assessment methods, genetic markers investigated, health outcomes assessed, and key findings related to gene-environment interactions. A detailed overview of these extracted data, presented in Supplementary Table S1, provides a comprehensive summary of the key characteristics of the included studies, enabling comparison and identification of research gaps. A narrative synthesis of the findings were then conducted to map the existing literature and identify key themes and research gaps [28]–[30],[33],[34].

### 2.6. Quality assessment of included studies

To strengthen the methodological rigor of our review, we conducted a formal quality appraisal of all 16 included full-text articles. Given the variety of study designs, we employed appropriate assessment tools tailored to each design type:

- The 13 prospective cohort studies were assessed using the Newcastle-Ottawa Scale (NOS) [35].
- The 1 cross-sectional study was evaluated using a modified version of NOS tailored for cross-sectional designs.
- The 1 meta-analysis was assessed narratively using AMSTAR 2 criteria, which were widely accepted for systematic reviews and meta-analyses [36].
- The 1 molecular-epigenetic cohort study, although fundamentally prospective in design, was evaluated using the JBI Critical Appraisal Checklist for Cohort Studies due to its integration of biological, genetic, and epigenetic data [37].

## 3. Results

**Figure 2.**
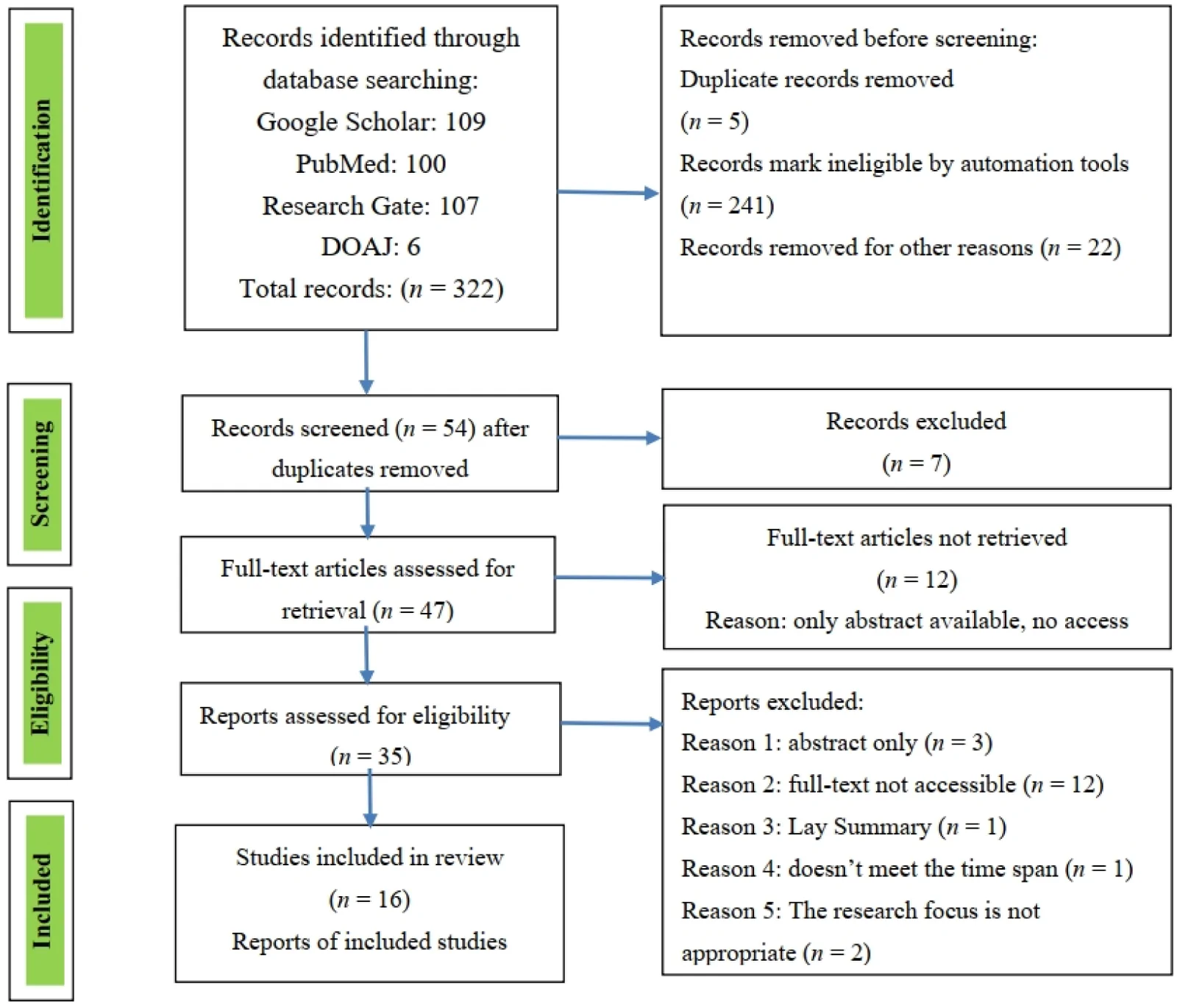
PRISMA-ScR flow diagram.

A total of 322 records were identified through database searching (PubMed *n* = 100, Google Scholar *n* = 109, Research Gate *n* = 107, and DOAJ *n* = 7). After removing duplicates (*n* = 5), 315 records underwent title and abstract screening. Of these, 283 were excluded as they did not meet the inclusion criteria (e.g., not focused on genetic susceptibility to air pollution, review articles, or non-human studies). A total of 35 full-text articles were assessed for eligibility, and 19 were further excluded due to methodological concerns (e.g., lack of a clear methodology, or focus on non-PM_2.5_ pollutants), or lack of investigation of gene-environment interaction). Finally, 16 studies met all inclusion criteria and were included in this scoping review (Figure 2).

### 3.1. Study characteristics

This scoping review included 16 studies investigating the interplay between genetic susceptibility and air pollution, particularly PM_2.5_, on various health outcomes. A diverse range of study designs were employed, including 1 cross-sectional study, 14 prospective cohort studies, 1 meta-analysis of cohort studies, and 1 Mendelian Randomization study. This heterogeneity in study design is typical in a scoping review, aiming to map the available evidence regardless of methodological rigor [38]–[53]. Only one study employed Mendelian Randomization analysis [54]–[56] as its core methodological approach.

Most studies focused on adult populations, with a reported age range spanning from 37 to 73 years. Geographically, the research was predominantly conducted in Europe (*n* = 12), with one study encompassing both Europe and North America (*n* = 1), and a smaller number conducted in Asia (*n* = 3). This geographical distribution highlights a potential gap in research from other regions. Furthermore, 15 out of the 16 studies investigated the combined effects of particulate matter (PM_2.5_ and/or PM_10_), nitrogen dioxide (NO_2_), and nitrogen oxides (NO_x_). Only one study, Gruzieva et al. (2016) [38], focused solely on prenatal NO_2_ exposure. This pattern suggests that PM_2.5_ and NO_2_ are dominant environmental factors in the studies and highlights the need for further exploration of NO_2_ exposure, particularly in its isolated form, to better understand its role in genetic susceptibility to diseases [39]–[53].

PM_2.5_ exposure was the most commonly assessed air pollutant, primarily using air quality monitoring data (*n* = 16). It should be noted that some studies used multiple methods for exposure assessment. Some studies utilized land-use regression models to estimate PM_2.5_ exposure based on spatial data and environmental characteristics, while others employed self-reported questionnaires focusing on residential location and daily activities. For instance, Huang et al. (2021) [39] and Gao et al. (2023) [52] used land-use regression models within the UK Biobank to estimate individual exposures. Li et al. (2023) [41] used land-use regression models in China. Air quality monitoring data typically involves measurements taken at fixed monitoring stations, providing information on ambient air pollution levels in specific locations. Land-use regression models, on the other hand, incorporate spatial data such as traffic density, land use types, and meteorological factors to create more refined estimates of pollution exposure at a finer spatial scale [39],[41],[52]. These methods have varying degrees of accuracy and may introduce different types of measurement error.

**Operational Definitions of Variables:** PM_2.5_ was most often defined as the annual average concentration at the participants' residential address. However, some studies used different averaging periods (e.g., 24-hour average) or considered specific sources of PM_2.5_ (e.g., traffic-related PM_2.5_). Health outcomes varied across studies, encompassing cardiovascular diseases (e.g., myocardial infarction, stroke), respiratory diseases (e.g., chronic obstructive pulmonary disease (COPD), lung cancer), and metabolic disorders (e.g., type 2 diabetes). This variability in outcome definitions should be considered when interpreting the findings.

**Exposure Measurement Methods (Further Details):** Studies using air quality monitoring data often linked participants' residential addresses to the nearest monitoring station. Land-use regression models incorporated geographic information system (GIS) data on traffic, land use, and topography. Self-reported questionnaires typically asked participants about their residential history, time spent outdoors, and proximity to pollution sources.

**Justification for Study Selection:** Mendelian Randomization studies were included because they provide stronger evidence for causal inference using genetic variants as instrumental variables, reducing the potential for confounding and reverse causation. Studies employing other designs, such as cohort studies, were included to provide a broader overview of the existing evidence base [49],[54]–[56].

Information on sex was consistently reported, with approximately equal representation of men and women across the studies. However, reporting on other demographic characteristics, such as ethnicity and socioeconomic status (SES), was less consistent. Where reported, SES was often categorized based on indicators such as education level, occupation, or income. Some researchers also considered other participant characteristics such as smoking status and pre-existing health conditions as potential confounders.

**Interventions or Moderating Factors:** Several studies investigated potential moderating factors such as genetic polymorphisms (as mentioned previously), dietary intake, and physical activity. For instance, Huang et al. (2021) examined whether the association between PM_2.5_ and lung function was modified by genetic variations in antioxidant enzymes [39].

Sixteen studies entailed the interactions of PM_2.5_, PM_10_, NO_2_, and NO_x_ on various gene polymorphisms associated with increased disease risk. These studies often examined specific gene variants known to be involved in pathways related to inflammation, oxidative stress, or DNA repair, which are mechanisms through which air pollution is thought to exert its effects. Mendelian Randomization studies were included to provide stronger causal evidence for the relationship between air pollution and health outcomes. Mendelian Randomization utilizes genetic variants as instrumental variables to assess the causal effect of an exposure (e.g., air pollution) on an outcome (e.g., disease risk), minimizing the influence of confounding factors [49],[54]–[56]. Only one meta-analysis of cohort studies specifically examined the relationship between NO_2_ exposure during pregnancy and cord blood DNA methylation. This meta-analysis synthesized data from multiple cohort studies to investigate the potential impact of prenatal NO_2_ exposure on epigenetic modifications in newborns [39].

**Brief Summary of Key Findings:** Overall, the studies consistently suggested a positive association between long-term exposure to air pollutants, particularly PM_2.5_, and adverse health outcomes, including cardiovascular and respiratory diseases. Some studies also found evidence of associations with metabolic disorders and other health outcomes. Researchers investigating gene-environment interactions provided evidence that genetic susceptibility can modify the effects of air pollution [38]–[53].

Some researchers used ge_no_typing to assess genetic susceptibility and data from air quality monitoring stations to measure PM_2.5_ exposure. The findings of the included studies generally suggested a positive association between long-term exposure to air pollutants, particularly PM_2.5_, and adverse health outcomes [38]–[53].

A detailed overview of the included studies, including author, year, location, study design, population and sample size, exposure variables, health outcome, and age range, is presented in Supplementary Table S1.

To assess the methodological rigor of the included studies, a formal quality appraisal was conducted using tools appropriate for each study design, as detailed in the Methods section (see Section 2.6). A comprehensive summary of the methodological quality assessment for all 16 included full-text articles is presented in Table 1. The results showed that most studies met high-quality criteria, supporting the reliability of the extracted findings. For a detailed breakdown of individual study scores and their respective quality assessments, please refer to Supplementary File S2.

#### 3.1.1. Overview and categorization of health outcome

The studies in this review report a diverse range of health outcomes associated with air pollution exposure, involving both physical and mental health conditions across different populations. These outcomes span multiple disease categories, highlighting the broad impact of pollutants such as PM_2.5_, PM_10_, NO_2_, and NO_x_ [38]–[53].

To facilitate analysis, the included studies were categorized into seven primary groups: Respiratory diseases, cardiovascular diseases, neurological and psychiatric disorders, cancers, autoimmune and inflammatory conditions, and other diseases. Cardiovascular and neurological conditions were the most frequently studied, with consistent associations reported for PM_2.5_, PM_10_, NO_2_, and NO_x_ exposure. Notable findings include stronger associations of air pollution exposure with coronary artery disease (Fu et al., 2023; Li et al., 2022) and major depressive disorder (Li et al., 2023) associated with these pollutants [41],[42],[48]. Additionally, autoimmune conditions such as inflammatory bowel disease (Chen et al., 2024) were linked to long-term exposure to PM_2.5_ and NO_x_ [49].

**Table 1.** Summary of methodological quality assessment of the included studies based on study design.

**No.**	**Study (First Author, Year)**	**Study Design**	**Quality Assessment Tool**	**Score/Result**	**Notes**
1	Gruzieva et al., 2016 [38]	Meta-analysis (Cohort Data)	AMSTAR 2	High Quality	Evaluated narratively using AMSTAR 2
2	Huang et al., 2021 [39]	Prospective Cohort	NOS	9/9	UK Biobank, lung cancer
3	Ma et al., 2024 [40]	Prospective Cohort	NOS	9/9	UK Biobank, AAA
4	Li et al., 2023 [41]	Prospective Cohort	NOS	9/9	UK Biobank, MDD
5	Fu et al., 2023 [42]	Prospective Cohort	NOS	9/9	Based on UK Biobank, CAD
6	Ma et al., 2024 [43]	Prospective Cohort	NOS	9/9	Stroke, robust adjustment
7	Liu et al., 2024 [44]	Prospective Cohort	NOS	9/9	Schizophrenia
8	Huang et al., 2024 [45]	Prospective Cohort	NOS	9/9	Parkinson's disease
9	Wang et al., 2022 [46]	Prospective Cohort	NOS	9/9	COPD + interaction lifestyle
10	Rhee et al., 2024 [47]	Prospective Cohort	NOS	9/9	Cardiovascular disease
11	Li et al., 2022 [48]	Prospective Cohort	NOS	9/9	PM_2.5_ and CAD
12	Chen et al., 2024 [49]	Molecular-Epigenetic Cohort	JBI Checklist (Cohort)	High Quality	Epigenetic focus, UK Biobank based
13	Wu et al., 2024 [50]	Prospective Cohort	NOS	9/9	Psoriasis
14	Zhang et al., 2024 [51]	Cross-Sectional	Modified NOS (Cross-Sectional)	9/10	High quality cross-sectional design
15	Gao et al., 2023 [52]	Prospective Cohort	NOS	9/9	Depression and anxiety
16	Zhang et al., 2024 [53]	Prospective Cohort	NOS	9/9	Dementia

Note: Abbreviations: AMSTAR 2, Assessment of Multiple Systematic Reviews-2 (A Measurement Tool to Assess Systematic Reviews 2); NOS, Newcastle-Ottawa Scale; JBI, Joanna Briggs Institute; AAA, Abdominal Aortic Aneurysm; MDD, Major Depressive Disorder; CAD, Coronary Artery Disease; COPD, Chronic Obstructive Pulmonary Disease; and PM_2.5_, Particulate Matter with a diameter ≤ 2.5 µm.

Having established the characteristics of the included studies and the methods used to assess exposure and outcomes, in the following section, we detail the methods used to assess genetic susceptibility and pollutant exposure before presenting the key findings related to gene-environment interactions [38]–[53].

##### 3.1.1.1. Respiratory diseases

Several studies focus on respiratory conditions, particularly in relation to particulate matter and nitrogen oxides:

- Wang et al. (2022): **Chronic obstructive pulmonary disease (COPD)** associated with PM_2.5_, PM_10_, NO_2_, and NO_x_ [46].

##### 3.1.1.2. Cardiovascular diseases

Air pollution exposure is strongly linked to various cardiovascular conditions:

- Ma et al. (2024): **Abdominal aortic aneurysm** [40].
- Fu et al. (2023): **Coronary artery disease (CAD)** [42].
- Rhee et al. (2024): **General cardiovascular diseases** [47].
- Li et al. (2022): **Coronary artery disease (CAD)** [48]

##### 3.1.1.3. Neurological and psychiatric disorders

Mental health and cognitive impairments are key areas of concern:

- Li et al. (2023): **Major depressive disorder** [41].
- Liu et al. (2024): **Schizophrenia** [44].
- Zhang et al. (2024): **Speed processing deficits** [51].
- Gao et al. (2023): **Depression and anxiety** [52].
- Zhang et al. (2024): **Dementia** [53].

##### 3.1.1.4. Cancer

A study reports a significant association between air pollution and lung cancer:

- Huang et al. (2021): **Lung cancer** [39].

##### 3.1.1.5. Autoimmune and inflammatory conditions

- Chen et al. (2024): **Ulcerative colitis** [49].
- Wu et al. (2024): **Psoriasis** [50].

##### 3.1.1.6. Epigenetic changes

Air pollution exposure, particularly in early life, has been shown to cause epigenetic changes, such as differential DNA methylation:

- Gruzieva et al (2016): **Differential offspring DNA methylation at CpG site** in cord blood newborns [38].

##### 3.1.1.7. Other diseases

Several studies have also linked air pollution to other health conditions:

- Ma et al. (2024): **Stroke** [43].
- Huang et al. (2024): **Parkinson's disease** [45].

#### 3.1.2. Methods of exposure and outcome assessment

In this scoping review, the methods used to assess air pollution exposure and health outcomes varied across studies, reflecting the diversity of study designs and populations.

##### 3.1.2.1. Exposure assessment

In this review, we categorized the approaches used to assess exposure to air pollution into three main groups:

**1) Air Quality Monitoring and Dispersion Models:** Exposure estimated from fixed-site monitoring data or government-provided dispersion models (e.g., DEFRA in the UK). These methods provide spatially resolved estimates of pollutants such as PM_2.5_, PM_10_, NO_2_, and NO_x_ [40],[44],[51].

**2) Land-Use Regression Models:** Several researchers (e.g., Huang et al., 2021) employed land-use regression (LUR) models to estimate individual-level exposures to air pollution. LUR models use spatial data on environmental and urban characteristics, such as traffic density, land use, and meteorological factors, to predict exposure to pollutants at a finer geographic scale. These models can provide more localized estimates of exposure, accounting for variation in pollution levels that may not be captured by monitoring stations [39],[41],[52].

**3) Self-Reported Questionnaires:** A few researchers included in this review also used self-reported questionnaires, asking participants about their residential history, time spent outdoors, and proximity to pollution sources. This method, while less accurate than air quality monitoring or LUR models, enabled researchers to estimate individual exposure based on participants' reported behaviors and locations [50].

**4) Satellite-based Approaches:** A limited number of studies estimated exposure using satellite-derived data, such as aerosol optical depth (AOD), often combined with meteorological and land-use variables through machine learning models to provide high-resolution estimates of ambient PM_2.5_ concentrations [48].

##### 3.1.2.2. Outcome assessment

**1) Health Outcomes:** A broad range of health outcomes were assessed across the studies, including respiratory diseases (e.g., COPD, and asthma), cardiovascular diseases (e.g., coronary artery disease, and myocardial infarction), neurological conditions (e.g., dementia, and depression), metabolic disorders (e.g., type 2 diabetes), and autoimmune/inflammatory diseases (e.g., ulcerative colitis). Each study defined and measured these outcomes differently, with some relying on clinical diagnoses, hospital records, or self-reported health conditions [38]–[53].

**2) Objective Health Measurements:** Many researchers used objective health measures, such as lung function tests, blood pressure readings, or biomarkers, to assess the impact of air pollution on various health conditions. These measurements provided more precise and quantifiable data compared to self-reported health information.

**3) Gene-Environment Interactions:** A subset of studies explored how genetic susceptibility modifies the impact of air pollution on health outcomes. These studies integrated genetic data (e.g., from genotyping or epigenetic analyses) with environmental exposure estimates.

Details of methodological examination of gene–environment interactions are provided in section (3.1.3).

#### 3.1.3. Gene–environment interaction analysis

To enhance transparency and methodological rigor, we examined how the included studies assessed gene–environment (GxE) interactions. All 16 studies investigated the modifying role of genetic susceptibility on the association between air pollution exposure and health outcomes. However, the methodological approaches varied.

Several researchers employed Cox regression models to estimate hazard ratios and to evaluate interaction effects [39]–[46],[48],[49],[53]. Among these, a subset formally tested additive interaction metrics, such as the Relative Excess Risk due to Interaction (RERI) and Attributable Proportion (AP), which provide insight into the biological synergy between genetic risk and environmental exposure [38],[39],[41],[42],[45],[48],[52]. Multiplicative interactions, expressed through interaction coefficients in Cox models, were also reported in some studies.

Only a subset of researchers formally tested gene–environment interactions, either through additive metrics (e.g., RERI, and AP) or multiplicative interaction terms. Several studies (e.g., Fu et al. and Rhee et al.) reported combined effect estimates without direct interaction testing, which may limit interpretability. We have reflected these methodological distinctions in Supplementary Table S3. To support methodological clarity in future research, we encourage adherence to established guidelines for GxE analysis, including the use of formal interaction testing and transparent reporting of effect modification approaches.

While most researchers did not apply formal multiple testing corrections (e.g., Bonferroni or false discovery rate), two studies, those by Gruzieva et al. (2016) and Zhang et al. (2024), did report correction procedures [38],[53]. However, the lack of correction in most studies may limit the interpretability of interaction findings in the presence of multiple comparisons. This issue is particularly relevant given the large number of exposures and genetic markers tested, which increases the chance of false-positive results.

A detailed summary of the interaction testing methods, effect sizes, p-values, and confidence intervals is provided in Supplementary Table S3. To improve visibility and address reviewer concerns, we have clarified key methodological features in this section and will consider integrating selected elements of Supplementary Table S3 into the main manuscript if appropriate.

To complement Supplementary Table S3, which details the interaction testing methods used in each study, Table 2 summarizes key methodological characteristics of the included studies, focusing on the statistical approaches used to evaluate gene–environment interactions, the type of interaction tested (multiplicative or additive), the significance of interaction terms (e.g., p-values), and the application of multiple testing corrections. This structured summary enhances methodological transparency and supports interpretation of the findings by distinguishing between formal and informal testing strategies.

**Table 2.** Overview of formal and informal testing methods, interaction type, and multiple testing correction in gene–environment interaction studies.

**No**	**Study**	**Formal Interaction**	**Informal Interaction**	**Interaction Type**	**Interaction Significance and Strength**	**Multiple Testing Correction**
1	Gruzieva et al., 2016 [38]	Not Available	Narrative Synthesis	Epigenetic	Not reported (unclear)	False Discovery Rate (FDR)
2	Huang et al., 2021 [39]	Cox proportional hazard models, RERI, AP	-	Multiplicative Positive Additive	Not reported (unclear)	Not Reported
3	Ma et al., 2024 [40]	Cox proportional hazard models, RERI, AP	-	Multiplicative Positive Additive	Not reported (unclear)	Not Reported
4	Li et al., 2023 [41]	Cox proportional hazard regression models (*p*-interaction and Hazard Ratio)	Stratified Analysis	Multiplicative	PM_2.5_: *p* = 0.036 PM_10_: *p* = 0.025 NO_2_: *p* = 0.030 (**Significant**) NO_x_: *p* = 0.080 (**Not Significant**)	Not Reported
5	Fu et al., 2023 [42]	Cox proportional hazard regression models (*p*-interaction and Hazard Ratio), RERI, AP	Subgroup HR Comparison (by PRS)	Multiplicative Positive Additive	All *p*-interaction > 0.05 (**Not Significant**)	Not Reported
6	Ma et al., 2024 [43]	Cox proportional hazard regression models (*p*-interaction and Hazard Ratio), RERI, AP, Aalen Additive Hazard Model	-	Multiplicative Positive Additive	Not reported (unclear)	Not Reported
7	Liu et al., 2024 [44]	Cox proportional hazard regression models (*p*-interaction and Hazard Ratio)	Stratified Analysis	Multiplicative	PM_2.5_: *p* = 0.48 (**Not Significan** PM_10_: *p* = 0.79 (**Not Significant**) NO_2_: *p* < 0.07 (**Not Significant**)	Not Reported
8	Huang et al., 2024 [45]	Cox proportional hazard regression models (*p*-interaction and Hazard Ratio)	Stratified Analysis	Multiplicative	Not reported (unclear)	Not Reported
9	Wang et al., 2022 [46]	Cox proportional hazard regression models (*p*-interaction and Hazard Ratio), RERI, AP	-	Multiplicative Positive Additive	All *p*-interaction > 0.05 (**Not Significant**)	Not Reported
10	Rhee et al., 2024 [47]	Not Reported	Visual Trend and Stratified HR	Descriptive only	Not reported (unclear)	Not Reported
11	Li et al., 2022 [48]	Cox proportional hazard regression models (*p*-interaction and Hazard Ratio)	Narrative Interpretation	Multiplicative	*p*-interaction < 0.001 (**Strong Evidence**)	Not Reported
12	Chen et al., 2024 [49]	Cox proportional hazard regression models (*p*-interaction and Hazard Ratio), RERI, AP	-	Multiplicative Positive Additive	*p*-interaction (multiplicative) = 0.275 (**Not Significant)** *p*-interaction (additive) = 0.00123 (**Significant**)	Not Reported
13	Wu et al., 2024 [50]	Not Available	Narrative Association	Informal	PM_10_: *p* = 0.002 (**Significant**), PM_2.5_: *p* = 0.105 (**Not Significant**), NO_2_: *p* = 0.051 (**Not Significant**) PM_10_ (Additive): **Not Reported.**	Not Reported
14	Zhang et al., 2024 [51]	Not Available	Stratified Analysis	Informal	Not reported (unclear)	Not Reported
15	Gao et al., 2023 [52]	Not Reported	Synergistic/ enhancing effect (Gene Environment Interaction)	Multiplicative	Not reported (unclear)	Not Reported
16	Zhang et al., 2024 [53]	Cox proportional hazard models (*p*-interaction and Hazard Ratio), RERI, AP	-	Multiplicative Positive Additive	HR interaction term reported (exact p not stated); RERI, and AP stated.	HMP (harmonic mean *p*-value); PFWE & PFDR in imaging

Note: Abbreviations: FDR, False Discovery Rate; RERI, Relative Excess Risk due to Interaction (the proportion of disease among those with both the exposure and the genotype that is attributable to their interaction); AP, Attributable Proportion due to Interaction (the proportion of disease in the population that is attributable to the interaction between the exposure and genotype); HR, Hazards Ratio; PM_2.5_, Particulate Matter with a diameter ≤ 2.5 µm; PM_10_, Particulate Matter with a diameter ≤ 10 µm; NO_2_, Nitrogen Dioxide; NO_x_, Nitrogen Oxides; HMP, Harmonic Mean *p*-value; PFWE, Permutation-based Family-Wise Error rate; and PFDR, Permutation-based False Discovery Rate. **Formal interaction testing** includes **regression-based interaction terms** (e.g., *p*-interaction), as well as measures on the **additive scale** such as RERI (Relative Excess Risk due to Interaction), AP (Attributable Proportion), and the Synergy Index. **Informal interaction testing** includes subgroup or stratified analysis, visual inspection of effect modification across strata, or narrative/descriptive comparisons without formal statistical interaction terms. **Interaction type** refers to whether the interaction was evaluated on the **additive scale**, **multiplicative scale**, or only through **informal exploration** (without formal statistical testing). **Multiple testing correction** refers to statistical methods used to adjust for the number of tests performed, such as Bonferroni correction or False Discovery Rate (FDR) control, and Harmonic Mean *p*-value (HMP).

### 3.2. Methods to assess genetic susceptibility and pollutant exposure

In this section, we describe the specific methods used within the 16 included studies to assess genetic susceptibility and pollutant exposure. We focus on *how* these measurements were implemented in the context of the reviewed literature, rather than providing a general overview of these methods.

#### 3.2.1. Assessment of genetic susceptibility

Among the 16 articles reviewed, 14 focused on genetic susceptibility, 1 examined epigenetic modification, and 1 study entailed both genetic susceptibility and epigenetic modification. Table 3 summarizes the focus of these articles.

**Table 3.** Summary of study focus.

**Study Type**	**Number of Articles**
Genetic Susceptibility	13
Epigenetic Modification	2
Both Genetic and Epigenetic	1

Note: Table 3 provided a breakdown of the types of studies included in this review.

After assessing general genetic susceptibility, we also explored gene-environment (GxE) interactions; how genetic factors may modify the health effects of air pollution exposure. Table 4 presents an overview of these studies, focusing on the use of **genotyping** or **DNA methylation** methodologies.

**Table 4.** Overview of Studies on GxE Interactions Using Genotyping or DNA Methylation.

**Study**	**Population**	**Methodology**	**Exposure (Pollutant)**	**Outcome (Disease)**	**Type of analysis**	**Key findings**
Gruzieva et al., 2016 [38]	Newborns, child-aged 4 and 8 from European and North America (*n* = 1508 newborns, *n* = 733 at age 4, *n* = 786 at age 8).	DNA methyla-tion (Epige-nome-Wide),meta-analysis of cohort study.	Prenatal NO_2_ exposure.	Altered DNA methylation at CpG sites in FAM13A and NOTCH4.	Epigenetic Modification.	Early life epigenetic markers link to respiratory disease later.
Huang et al., 2021 [39]	455,974 participants aged 40–69 years (UK Biobank).	PRS calculation based on 18 SNPs In lung cancer; Land-Use Regres-sion (LUR) models; Analytical cohort study.	Ambient air pollution (PM_2.5_, NO_2_, PM_10_, NO_x_).	Lung cancer incidence.	Genetic risk interaction/ Environmental exposure Statistical ( Cox proportional Hazard models); RERI, AP.	Air pollution exposure significantly associated with higher likelihood of lung cancer (63% higher), particularly among individuals with high genetic susceptibility.
Ma et al., 2024 [40]	449,463 participants aged 37–73 years from the UK Biobank.	Polygenic risk score (PRS) based on 31 SNPs; Air pollution exposure data; cohort study.	Long-term exposure to air pollutants (PM_2.5_, PM_10_, NO_2_, NO_x_).	Incidence of Abdominal Aortic Aneurysm (AAA).	Genetic susceptibility, Statistical (Cox proportional hazard models).	Long-term air pollution exposure is associated. with increased likelihood of AAA; genetic risk (PRS) also plays a role in susceptibility.
Li et al., 2023 [41]	354,897 participants aged 37–73 years from the UK Biobank.	Polygenic risk score (PRS), using 17 MDD-associated genetic loci (17 SNPs); Land-Use Regression (LUR) models; cohort study.	Long-term exposure to air pollutants (PM_2.5_, PM_10_, NO_2_, NO_x_).	Incidence of Major Depressive Disorder.	Genetic susceptibility, Statistical (Cox proportional hazard models).	Long-term air pollution exposure is associated with increased likelihood of MDD; genetic risk (PRS) also plays a role in susceptibility.
Fu et al., 2023 [42]	407,470 participants aged 40–69 years from the UK Biobank.	CAD genomewide association meta-analysis with-out the UK Biobank population with 40 SNPs; cohort study.	Long-term exposure to air pollutants (PM_2.5_, PM_10_, NO_2_, NO_x_).	Incidence of Coronary Artery Disease (CAD).	Genetic susceptibility, Statistical (Cox proportional hazard models, RERI, AP).	Long-term air pollution exposure is associated with increased likelihood of CAD; genetic risk (PRS) also plays a role in susceptibility.
Ma et al., 2024 [43]	452,196 partici years from the UK Biobank.	Polygenic risk score (PRS) Calculation With 71 SNPs; cohort study	Long-term air pollutants (PM_2.5_, PM_10_, NO_2_, NO_x_).	Incidence of Stroke, Ischemic Stroke, Hemorrhagic Stroke.	Genetic susceptibility, Statistical (Cox proportional hazard models).	Long-term air pollution exposure is associated with increased likelihood of stroke. genetic risk (PRS) also plays a role in susceptibility.
Liu et al., 2024 [44]	485,288 participants) aged 37–73 years from the UK Biobank.	Genome-wide association studies; Polygenic risk score (PRS) calculation, cohort study.	Long-term air pollutants (PM_2.5_, PM_10_, NO_2_, NO_x_).	Incidence of schizophrenia.	Genetic susceptibility, Statistical (Cox proportional hazard models).	Long-term air pollution exposure is associated with stronger association with schizophrenia; genetic risk (PRS) also plays a role in susceptibility.
Huang et al., 2024 [45]	over 312,000 participants. Average aged 57 years.	Polygenic risk score (PRS) Calculation; cohort study.	Long-term air pollutants (PM_2.5_, PM_10_, NO_2_, NO_x_).	Incidence of Parkinson's Disease (PD).	Genetic susceptibility, Statistical (Cox proportional hazard models).	Long-term air pollution exposure is associated with higher odds of Parkinson's Disease (PD); genetic risk (PRS) also plays a role in susceptibility.
Wang et al., 2022 [46]	452,762 participants aged 37–73 years from the UK Biobank.	Genotyping by Affymetrix Research Services Laboratory in 106 sequential batches of ap Prox. 4,700 samples; selected 22 SNPs associated with COPD; Weighted genetic risk score calculation; cohort study.	Long-term air pollutants (PM_2.5_, PM_10_, NO_2_, NO_x_).	Incidence of chronic obstructed Pulmonary Disease (COPD).	Genetic susceptibility, Statistical (Cox proportional hazard models).	Long-term air pollution exposure is associated with stronger likelihood of COPD; Weighted genetic risk also plays a role in susceptibility.
Rhee et al., 2024 [47]	249 082 participants aged 40–69 years.	Genotyping of 807411 SNPs; Polygenic risk score (PRS) calculation, cohort study.	Long-term air pollutants (PM_2.5_, PM_10_, NO_2_, NO_x_).	Incident CardioVascular Disease (CVD).	Genetic susceptibility, Statistical (Cox proportional hazard models).	Long-term air pollution exposure is associated with increased odds of Cardiovascular Disease (CAD); genetic risk (PRS) also plays a role in susceptibility. No significant interactions between genetic risk and PM2.5 exposure on cardiovascular death or CVD events.
Li et al., 2022 [48]	41,149 participants from China-PAR.	Polygenic risk score (PRS) calculation based on 540 genetic variants; cohort study.	Long-term air pollutants (PM_2.5_, PM_10_, NO_2_, NO_x_).	Incidence of Coronary Artery Disease (CAD).	Genetic susceptibility, Statistical (Cox proportional hazard models).	Long-term air pollution exposure is associated with increased odds of Coronary Artery Disease (CAD); polygenic risk score (PRS) also plays a role in susceptibility.
Chen et al., 2024 [49]	453,919 individuals aged 40–69 years; White European descent.	DNA methylation alterations at CXCR2 and sites within the MHC class III region.	Long-term air pollutants (PM_2.5_, PM_10_, NO_2_, NO_x_).	Incidence of Ulcerative colitis (UC).	Epigenetic Modification; Statistical (Cox proportional hazard models; epigenetic Mendelian Randomization approach).	Higher exposures to NOx, NO2, PM2.5 and combined air pollution score were associated with incident UC but not CD.
Wu et al., 2024 [50]	474,055 participants aged 40–69 yaers.	Polygenic risk Score (PRS) Calculation; cohort study.	Long-term air pollutants (PM_2.5_, PM_10_, NO_2_, NO_x_).	Incident Psoriasis.	Genetic susceptibility, Statistical (Cox proportional hazard models); RestricTed Cubic Spline Models; sensitivity analyses.	There was an interaction between air pollution and genetic suscptibibility in relation to psoriasis.
Zhang et al., 2024 [51]	522 healthy participants aged 40–69 years living in Beijing	Polygenic risk score (PRS) calculation; DNA methylation; cross-sectional study.	Long-term air pollutants (PM_2.5_, PM_10_, NO_2_, NO_x_).	Depression on processing speed.	- Genetic susceptibility: PRS; - Epigenetic Modification: DNA Methylation; - Genetic Modification (of EnVironmental Effects): GLM, and PLSR.	Air pollution may be associated with an increased likelihood of cognitive impairment in individuals genetically predisposed to dePression. The article does not provide specific effect sizes, but it describes the direction of the interaction (worsening effect with combined exposure and higher polygenic risk score).
Gao et al., 2023 [52]	502,536 participants from the UK Biobank, recruited in 2006–2010.	Polygenic risk score calculation (Depression: 37 SNPs Anxiety: 9 SNPs); Land-Use Regression (LUR) models; cohort study.	Long-term air pollutants (PM_2.5_, PM_10_, NO_2_, NO_x_).	Risk of Depression and Anxiety.	Genetic susceptibility, Statistical (Cox proportional hazard models).	Elevated levels of the five air pollutants were associated with higher odds of mental disorders at baseline.
Zhang et al., 2024 [53]	401,244 participants aged 40–69 years.	This article used genotyping data in2 ways: * Targeted genotyping: To get the APOE ε4 status. * Genome-wide genotyping: As the basis for calculating a PRS that incorporates many genetic variants associated with the outcome of interest (likely dementia or related traits).	Long-term air pollutants (PM_2.5_, PM_10_, NO_2_, NO_x_).	Incident Demensia.	Genetic susceptibility, Statistical (Cox proportional hazard models and Restricted Cubic Spline Regression).	Joint exposure to multiple air pollutants is associated with higher odds of dementia, especially among individuals with high genetic susceptibility.

Note: Abbreviations: GxE: Genotype by Environment; DNA: Deoxyribonucleic Acid; NO_2_: Nitrogen Dioxide; CpG: Cytosine-phosphate-Guanine; *FAM13A*, *NOTCH4*: Specific gene names involved in various biological processes (Further explanation could be provided in the main text if relevant to the study's focus). *Italicized gene names indicate standard gene nomenclature*; PRS: Polygenic Risk Score; LUR: Land-Use Regression; PM_2.5_: Particulate Matter with a diameter of 2.5 micrometers or less; PM_10_: Particulate Matter with a diameter of 10 micrometers or less; GLM, General Linear Model; and PLSR, Partial Least Squares Regression.

Table 4 provides an overview of studies on gene-environment interactions (GxE) using genotyping or DNA methylation. The studies listed highlight how genetic factors may influence the health outcomes of air pollution exposure, with a particular focus on epigenetic modifications like DNA methylation at specific CpG sites.

The assessment of genetic susceptibility in the included studies primarily focused on identifying specific genetic variants associated with increased risk of adverse health outcomes related to air pollution exposure. Many studies aimed to explore how genetic differences could modify the harmful effects of pollutants like PM_2.5_, PM_10_, NO_2_, and NO_x_ on health outcomes.

- **Genotyping Methods Used in Included Studies:** Most researchers employed genotyping techniques, with **SNP arrays** being the most common method (*n* = 14). These arrays enabled the detection of a wide range of single nucleotide polymorphisms (SNPs) across multiple genes. **Illumina Human Omni Express arrays** were utilized in some studies to assess SNPs related to oxidative stress and inflammatory pathways. Additionally, **PCR-based genotyping** methods, such as **TaqMan assays**, were used in a few studies to investigate specific candidate genes linked to air pollution-related health effects. Only a smaller number of studies (*n* = 2) employed **whole-genome sequencing (WGS)** to explore broader genetic variations, although this method was applied in a limited number of participants due to cost and technical constraints.
- **Candidate Genes and Genome-Wide Association Studies (GWAS)**: A combination of **candidate gene** approaches (*n* = 8) and **GWAS** (*n* = 7) were used in these studies to explore the genetic basis of susceptibility to air pollution-related health risks. Candidate gene studies often targeted well-known genes involved in inflammation or detoxification. In contrast, **GWAS** enabled the identification of novel genetic variants associated with exposure to pollutants.
- **Gene-Environment Interactions**: Several researchers in this review focused on **gene-environment interactions**, which investigate how genetic susceptibility can modify the health effects of air pollution exposure. In these studies, genetic data were typically obtained from blood, saliva, or buccal samples, and air pollution exposure was assessed through monitoring data or Land Use Regression (LUR) models [39],[41],[52]. Notably, the studies by Zhang et al. (2024) employed genotyping methods to examine the role of genetic polymorphisms in genes such as APOE ε4, FRMD8, DDX1, DNMT3L, MORC1, and TGM2 which are involved in specific biological pathways relevant to air pollution exposure such oxidative stress, neuroinflammation, and epigenetic regulation. These researchers found that certain genetic variants significantly influenced the association between air pollution exposure and incident Dementia [53].
- **Data Analysis and Quality Control**: Rigorous data analysis methods were employed across the studies to ensure the accuracy of genetic susceptibility results. Standard quality control measures, including filtering based on **minor allele frequency**, **call rates**, and testing for **Hardy-Weinberg equilibrium**, were commonly used to minimize errors. These procedures ensured that the genotyping data were reliable for assessing the associations between genetic variants and health outcomes [47].

#### 3.2.2. Assessment of pollutant exposure

The assessment of pollutant exposure in the included studies predominantly relied on environmental monitoring, modeling techniques, and personal exposure measurements to estimate the levels of air pollution to which study participants were exposed.

- **Environmental Monitoring and Air Quality Data**: A common method used in the studies was to obtain air quality data from government or environmental monitoring stations. These stations typically provide reliable data on the concentrations of pollutants, such as PM_2.5_, PM_10_, NO_2_, and NO_x_, at specific geographic locations. For example, several researchers (e.g., Zhang et al., 2024) utilized data from national or regional monitoring stations to estimate exposure for large cohorts. These data were often combined with residential or work addresses to estimate long-term exposure levels [51].
- **Land Use Regression (LUR) Models**: Many researchers (e.g., Huang et al., 2021, Li et al., 2023, Fu et al., 2023; and Gao et al., 2023) employed land use regression (LUR) models to predict pollutant levels in areas where direct monitoring data were not available. LUR models are particularly useful in estimating spatial variation in air pollution exposure by integrating geographical data, land use patterns, and other environmental factors. These models were applied to derive individual-level exposure estimates based on participants' residential locations. Different LUR models were used across studies, with varying levels of complexity and input data [39],[41],[42],[52].
- **Modeling Approaches**: Some studies employed advanced modeling approaches, including **dispersion models** and **satellite-based models**, to estimate air pollution exposure. For example, several studies based on the UK Biobank (e.g., Ma et al., 2024 [40]; Liu et al., 2024 [44]; Wu et al., 2024 [50]; Zhang et al., 2024 [53]) used **DEFRA air dispersion models** with a 1 × 1 km resolution to assign annual average pollutant concentrations to participants' residential addresses. In addition, Li et al., 2022 [48] applied a **satellite-based model** that combined aerosol optical depth (AOD) data with meteorological and land-use information using machine learning algorithms to estimate fine-scale PM_2.5_ exposure. These modeling approaches are particularly valuable in regions without dense monitoring station coverage.
- **Exposure Duration and Temporal Patterns**: Most studies evaluated long-term exposure (e.g., chronic exposure over years), but a few focused on short-term or acute exposure in relation to specific health outcomes (e.g., respiratory exacerbations or cardiovascular events). However, seasonal variations or temporal patterns of exposure were generally not explored in detail.
- **Exposure-Response Assessment**: Many studies included an exposure-response analysis to explore the relationship between pollutant levels and specific health outcomes. These studies often adjusted for confounding factors such as age, gender, socioeconomic status, and pre-existing health conditions to determine the strength and consistency of the exposure-response relationship [39],[41],[47].

**In summary**, the assessment of pollutant exposure in the reviewed studies utilized a combination of monitoring data and modeling techniques (including LUR, dispersion models, and satellite-based approaches). None of the included studies used personal exposure monitoring devices. The methodologies employed provided valuable insights into the health effects of air pollution by offering both spatially and temporally accurate exposure estimates.

#### 3.2.3. Integration of genetic and exposure assessments

The integration of genetic and exposure assessments is essential for understanding the complex interactions between genetic susceptibility and environmental exposures such as air pollution. In this section, we describe how researchers in this review combined genetic and environmental exposure data to examine gene-environment interactions (GxE), providing a deeper understanding of how genetic factors influence the effects of air pollution on health outcomes.

- **Stratified Analysis:** Some researchers in this review employed stratified analysis, where participants were divided into subgroups based on specific genetic variants to assess whether the effects of exposure differed between these subgroups. While not all studies used this approach, stratified analysis is commonly used to identify gene-environment interactions. For example, researchers have focused on polymorphisms in genes like GSTP1, involved in detoxification pathways, to explore how genetic variation might influence the response to air pollution. This approach provides deeper insights into how genetic factors can modify the health impacts of air pollution exposure [44],[45],[51].
- **Interaction Terms in Regression Models:** Statistical models (e.g., linear regression, and logistic regression) are used to test for the interaction between genetic variants and exposure variables. An interaction term is included in the model to assess whether the effect of exposure differs depending on genotype [39]–[46],[48],[49],[53].
- **Gene-Environment Interaction (GxE):** Gene–environment interaction (GxE) occurs when the impact of environmental exposure, such as air pollution, on health outcomes varies according to an individual's genetic profile. Among the 16 included studies, several explicitly tested GxE interactions using either multiplicative interaction terms in regression models or stratified analyses based on genetic risk categories (e.g., polygenic risk scores). These studies demonstrated that genetic susceptibility can modify the relationship between exposure to pollutants (e.g., PM_2.5,_ and NO_2_) and outcomes such as cardiovascular disease, major depressive disorder, or stroke. For example, some studies reported significantly greater adverse effects of air pollution among individuals in the highest tertile of genetic risk compared to those at lower risk [41],[42],[44],[46],[48]–[50].

In summary, the integration of genetic and exposure assessments using methods such as stratified analysis, regression models with interaction terms, GWIS, and consideration of gene-environment correlations provides valuable insights into how genetic susceptibility influences the health effects of air pollution. These approaches enhance our understanding of gene-environment interactions and are crucial for advancing precision medicine, where interventions can be tailored based on an individual's genetic profile and environmental exposures.

**Note on Supplementary Materials**: Due to the extensive nature of the data presented, Supplementary Table S4 provides a detailed summary of the key findings, conclusions, and limitations of the included studies. To ensure the flow and readability of the main text, this table has been moved to the Supplementary Materials section. Readers can refer to Supplementary Table S4 in the supplementary materials for a comprehensive overview of the studies included in this review.

#### 3.2.4. Gene-environment interactions

A detailed analysis of gene-environment interactions was conducted to explore how genetic predisposition modulates the health effects of air pollution. In Supplementary Table S3, we summarize the interactions between genetic markers and environmental exposures, such as PM_2.5_, PM_10_, NO_2_, and NO_x_, across multiple health outcomes, including cardiovascular diseases, respiratory conditions, and mental health disorders.

Key findings include:

- Significant interactions between specific genetic polymorphisms and pollutant exposure levels, with the strongest effects observed for cardiovascular diseases and mental health disorders.
- Variations in effect sizes (e.g., odds ratios, and hazard ratios) highlight the heterogeneity in genetic susceptibility to air pollution exposure across populations.
- Specific metrics such as Relative Excess Risk due to Interaction (RERI) and Attributable Proportion (AP) underscore the additive effects of genetic predisposition and environmental exposures on disease risk.

This table provides a comprehensive overview of the statistical evidence supporting the modifying role of genetic susceptibility in health outcomes associated with air pollution.

## 4. Discussion

The complex interplay between genetic predisposition and environmental exposures has emerged as a key area of research in understanding disease risk and health disparities. This review contributes to the growing body of literature by examining gene-environment interactions in the context of air pollution and their impact on various health outcomes [8],[35],[57].

### 4.1. Regarding the association between genetic predisposition and air pollution exposure

The interaction between genetic predisposition and environmental factors, such as air pollution, has garnered increasing attention in recent years due to its potential impact on disease risk. Our findings contribute to this growing body of literature, highlighting the significant role that genetic susceptibility plays in modifying the effects of air pollution on health outcomes [11],[32],[38],[43],[48]–[50],[57]–[60].

The additive effects observed in individuals with both high genetic susceptibility and high exposure to air pollution align with prior studies suggesting that genetic factors may amplify the adverse health effects of environmental pollutants. Specifically, we found that individuals at higher genetic risk exhibited more pronounced health deterioration when exposed to higher levels of air pollution. This combined effect, where the interaction between genetic susceptibility and environmental exposure exceed the sum of their individual effects, is consistent with other studies emphasizing the exacerbating role of genetic factors in the harmful effects of environmental stressors [39],[48].

Furthermore, genetic predisposition appears to modify the impact of air pollution exposure across various diseases, including cardiovascular diseases (CVD), respiratory conditions, and mental health disorders. These findings underscore the critical role of gene-environment interactions in shaping health outcomes. A detailed summary of gene-environment interactions, including the effect sizes, p-values, and health outcomes, is provided in Supplementary Table S3.

### 4.2. Regarding disease-specific findings

In line with other studies, long-term exposure to pollutants such as PM_2.5_, NO_2_, and PM_10_ was significantly associated with a higher likelihood of various diseases (e.g., lung cancer, cardiovascular disease, and stroke), especially among individuals with higher genetic susceptibility [38]–[53].

Our results confirm that the combined effect of air pollution and genetic predisposition plays a critical role in the development of complex diseases, including mental health disorders (e.g., schizophrenia, and Major Depressive Disorder) and cardiovascular diseases (e.g., abdominal aortic aneurysms). For conditions like ulcerative colitis and psoriasis, our findings suggest that air pollution exposure may be a modifiable environmental contributor, particularly for those genetically predisposed. This highlights the potential for public health interventions to target these conditions by addressing environmental exposures, such as through improved air quality policies. Further details of these interactions are presented in Supplementary Table S3.

### 4.3. Implications for public health and precision medicine

These findings underscore the need for personalized approaches in environmental health, where genetic susceptibility should be considered when assessing the potential impact of air pollution exposure. Identifying individuals with high genetic susceptibility for specific diseases and high exposure to air pollution could help target interventions and preventive strategies more effectively. For example, individuals with genetic susceptibility to respiratory diseases might benefit from policies aimed at reducing air pollution exposure in urban areas. Public health strategies could include prioritizing air quality improvements in regions with high genetic vulnerability indices, or incorporating genotyping into early screening programs in pollution-heavy urban centers [49],[50],[58].

While this review does not provide in-depth methodological analysis of these tools, we emphasize their future relevance for advancing the field. Although none of the included studies employed integrative multi-omics or machine learning techniques, these emerging methodologies are increasingly recognized as powerful tools in precision environmental health. They hold promise for uncovering novel mechanistic pathways and enabling more accurate risk stratification based on complex gene-environment interactions [17],[18],[21],[29].

Specifically, multi-omics and machine learning could significantly improve our understanding of how genetic factors modulate responses to air pollution, providing insights that could refine health outcome predictions and support personalized prevention strategies [18]–[21],[29],[60],[61].

Although genome-wide interaction studies (GWIS) were not identified among the included studies, researchers should consider applying GWIS to detect novel loci involved in pollution-related health effects [29],[32],[58],[62]. In addition, gene–environment correlation (rGE), where certain genetic traits predispose individuals to environments with higher pollution exposure, was not addressed in the included studies but remains an important methodological consideration for future analyses [63],[64]. Experimental studies have also highlighted the relevance of mechanistic pathways, such as aryl hydrocarbon receptor (AhR) signaling in response to PM_2.5_ exposure, yet this pathway was not explored in the reviewed epidemiological literature. These mechanisms warrant further investigation to strengthen the biological plausibility of GxE associations [65],[66]. In addition, future studies employing toxicological or experimental approaches, such as in vivo or organoid models, are needed to explore mechanistic pathways (e.g., oxidative stress, inflammation, and epigenetic regulation), which would strengthen the biological plausibility of observed GxE associations.

The data in Supplementary Table S3 support the potential value of combining genetic and environmental risk profiling in public health efforts, particularly in identifying and protecting vulnerable populations. As such, future research that integrate genetic data **with high-resolution exposure models, epigenomics, and machine learning algorithms** could substantially enhance targeted prevention strategies [38]–[53].

### 4.4. Limitations and recommendations for future research

While most included studies relied on observational designs, our findings are limited by the inability to establish causality and may be affected by residual confounding, particularly in the assessment of genetic susceptibility and environmental exposure [54],[67]. Based on the current evidence, we provide several recommendations for future research directions.

While we acknowledge that 12 of the 16 included studies were conducted in European populations or used UK Biobank data, the implications of this geographic and ethnic skew deserve deeper discussion. The lack of representation from non-European ancestry groups raises concerns about the external validity and equity of current GxE findings, particularly in the context of global precision health efforts. Equity and diversity should be central considerations when translating GxE insights into public health strategies [68],[69]. Recent advances in interaction testing frameworks have made it more feasible to detect complex GxE effects across populations [70]. Future research must explicitly include underrepresented populations, both to validate current findings and to uncover population-specific interactions that may be masked in predominantly European datasets [71]. This approach will enhance the relevance and fairness of GxE-informed precision health interventions on a global scale.

Further studies should address these limitations by incorporating more accurate exposure data, such as personal monitoring of air pollution, and exploring gene-environment interactions in more diverse populations to enhance the generalizability of the results [8],[70]–[72].

One study included in this review, one by Chen et al. (2024), presents distinct methodological considerations. While described as a cohort study, its structure is more akin to a cross-sectional or nested case-control design, as it lacks precise temporal data on ulcerative colitis onset [49]. This weakens the temporal relationship and introduces potential for reverse causation, which may limit causal inference. To mitigate these limitations, the authors employed epigenetic analysis and Mendelian randomization as complementary methods to strengthen causal interpretation [54]–[56]. Nevertheless, the absence of longitudinal follow-up reduces its methodological comparability with the prospective cohort studies included in this review. Therefore, quality assessment was performed using the JBI checklist rather than the Newcastle-Ottawa Scale, which better aligns with the study's epigenetic and case-control framework [35],[37]. Future studies investigating gene–environment interactions in ulcerative colitis should aim to replicate these findings using longitudinal designs with clearer temporal sequencing and larger population-based samples.

In addition, future studies would benefit from utilizing **multi-omics** approaches and **machine learning** techniques to explore the mechanistic pathways that link air pollution exposure with epigenetic changes and genetic predisposition in the development of complex diseases. These technologies have been highlighted as powerful tools to advance exposome research and understand causal biological mechanisms [17]–[21]. Such approaches hold great promise in identifying new biomarkers and uncover complex, multifactorial interactions that might otherwise be missed. The section on emerging technologies such as AI and multi-omics could also be expanded in future research to provide more detailed elaboration on their potential applications in improving exposure modeling, identifying complex gene-environment interactions, and enhancing risk prediction [21].

Moreover, **longitudinal designs** with larger, multi-ethnic samples and **standardized exposure assessments** will improve the robustness of future findings and enable a more nuanced interpretation of gene–environment dynamics over time. Although causality cannot be definitively inferred from observational data, enhancing study design and incorporating mechanistic approaches, such as multi-omics and molecular exposomics, can substantially strengthen the evidence base and help clarify potential biological pathways [20],[21],[73]–[80].

Finally, disease-specific recommendations should be considered. For instance, prioritizing the development and validation of polygenic risk scores (PRS) for conditions such as stroke, where strong genetic signals have been identified (e.g., Ma et al., 2024) [43], may help refine individual-level susceptibility profiling and enable more targeted public health responses [73]–[80]. Furthermore, as the field progresses towards potential applications of genetic information in public health strategies, careful consideration must be given to the **ethical implications of genetic screening**. These include ensuring robust data privacy and security measures, obtaining informed consent, addressing the potential for genetic discrimination, ensuring equitable access and implementation, and promoting responsible interpretation and application of genetic risk profiles [81].

### 4.5. Mechanistic evidence supporting GxE effects

Researchers using animal models and organoid systems demonstrate that air pollution triggers molecular events such as ROS overproduction, mitochondrial dysfunction, and cytokine dysregulation, which may interact with genetic predispositions to exacerbate disease processes [82],[83]. For example, in vivo models have shown that particulate matter exposure leads to neuroinflammation and cognitive impairment via the NF-κB and Nrf2 signaling pathways, providing insight into mechanisms potentially relevant to mental health outcomes [84],[85]. Similarly, lung and cardiovascular organoid models have revealed pollutant-induced endothelial dysfunction and inflammatory responses that mirror pathways implicated in human genetic risk loci [86],[87].

A recent review highlights how organoid and animal-based approaches are increasingly used to uncover the cellular and molecular mechanisms linking environmental exposures with chronic disease phenotypes. These mechanistic insights are essential for interpreting GxE interactions and underscore the need for integrative frameworks that combine epidemiological, genetic, and experimental evidence in environmental health research [88],[89].

To provide biological plausibility to the epidemiological associations observed in this review, it is important to consider experimental studies that elucidate underlying mechanisms. Toxicological and in vivo models have consistently shown that exposure to air pollutants such as PM_2.5_, NO_2_, and diesel exhaust particles can induce oxidative stress, systemic inflammation, and epigenetic changes; pathways that are also implicated in the genetic susceptibility to complex diseases [90],[91].

## 5. Conclusions

This review underscores the critical role of gene-environment interactions in shaping health outcomes, particularly in the context of air pollution exposure. Our findings suggest that genetic susceptibility may modify the associations of air pollution across various diseases, including cardiovascular conditions, respiratory disorders, and mental health challenges. These results provide compelling evidence for the need to integrate genetic data into environmental health research, enhancing our understanding of the complex relationships between pollution exposure and disease risk.

Given the observational nature of the included studies, causal relationships cannot be definitively established. Nonetheless, the patterns identified across the reviewed literature point to potentially important gene–environment interactions that merit further investigation through mechanistic and experimental studies.

The implications of these findings extend beyond scientific research, emphasizing the development of precision public health strategies. Identifying individuals with heightened genetic risk can enable the development of targeted prevention strategies, such as localized air quality interventions or early screening efforts for at-risk populations. In parallel, these insights reinforce the need for broad efforts to reduce air pollution exposure as a population-wide preventive strategy.

To improve the applicability of these findings, we recommend prioritizing the development of polygenic risk scores (PRS) for diseases with strong and consistent GxE signals, particularly stroke, as highlighted in recent studies such as Ma et al. (2024) [43]. Furthermore, enhancing air pollution monitoring systems in rapidly urbanizing low- and middle-income countries (LMICs) is essential to address current data gaps and guide targeted public health interventions.

Researchers should also incorporate mechanistic studies, including those using organoid and in vivo models, to support the biological plausibility of GxE effects. These experimental approaches can help elucidate key pathways such as oxidative stress, inflammation, and epigenetic modifications, thereby strengthening the interpretation of epidemiological associations.

Finally, to ensure the equity and global relevance of GxE research, future studies must include more diverse populations beyond those of European ancestry. By integrating genetic, environmental, and mechanistic evidence, future precision health strategies can be more effectively tailored to protect high-risk individuals and address the growing global burden of pollution-related diseases.

## Use of AI tools declaration

The author declare he has not used Artificial Intelligence (AI) tools in the creation of this article.


