# Supplementary material for: Exploring genetic susceptibility to air pollution and its implications for disease risk and precision health: A scoping review
Source: AIMS Public Health. 2025 Sep 11;12(3):916–51. doi: 10.3934/publichealth.2025046 (PMC12538249; doi:10.3934/publichealth.2025046)
Supplement: Supplementary file 1 [file publichealth-12-03-046-s001.pdf]

AIMS Public Health

AIMS Public Health, 12(3): 916–951.
DOI: 10.3934/publichealth.2025046
Received: 14 April 2025
Revised: 05 June 2025
Accepted: 16 June 2025
Published: 11 September 2025

https://www.aimspress.com/journal/aimsph

Review

Exploring genetic susceptibility to air pollution and its implications for disease risk and precision health: A scoping review

Hari Krismanuel*

Faculty of Medicine, Universitas Trisakti, West Jakarta, DKI Jakarta, Indonesia

* **Correspondence:** Email: hari_krismanuel@trisakti.ac.id.

Supplementary

Table S1. Characteristics of studies examining the association between air pollution and health outcomes.

Author & year	Location	Study Design	Population and Sample size	Exposure/ Variables	Health Outcome	Age range
Gruzieva et al., 2016 [38]	European and North American	Meta-analysis of cohort studies	Newborns, children aged 4 and 8 from European and North American cohorts (n = 1508 newborns, n = 733 at age 4, n = 786 at age 8)	NO2 exposure at residential addresses during pregnancy	DNA methylation in mitochondria related genes; methylation and expression of anti-oxidant and anti-inflammatory genes (CAT, TPO)	aged 4 and 8
Huang et al., 2021 [39]	UK Biobank/ UK	Analytical cohort study (using UK Biobank data)	455,974 participants in UK Biobank (53% women) with no previous history of cancer	Concentrations of PM (PM2.5, PMcoarse, PM10), NO2, and NOx estimated using land-use regression models	Incidence of lung cancer	40–69 years
Ma et al., 2024 [40]	UK Biobank/ 22 centres across the UK	Cohort study (using UK Biobank data)	449,463 participants from the UK Biobank	Long-term exposure to PM2.5, PM10, NO2, and NOx measured over time	Incidence of Abdominal Aortic Aneurysm (AAA)	37–73 years
Li et al., 2023 [41]	UK Biobank/ 22 centers in urban areas of England, Wales, and Scotland	Prospective cohort study (using UK Biobank data)	354,897 participants aged 37–73 years from the UK Biobank	Annual average concentrations of PM2.5 PM10, NO2, and NOx estimated using a Land Use Regression model	Incidence of Major Depressive Disorder (MDD)	37–73 years
Fu et al., 2023 [42]	UK Biobank (approximately 487,507 participants recruited across the UK .at baseline from 2006 to 2010.	Prospective cohort study.	407,470 participants were investigated the relationship of PM, genetic factors, and CAD, and 438,736 in the NO group. These participants were free of CAD at baseline (the start of the study).	Long-term exposure to air pollutants, including: (PM2.5), (PM10), Nitrogen dioxide (NO2), Nitrogen oxides (NOx).<br>Genetic Variable: Polygenic risk score (PRS) for CAD, representing an individual's genetic susceptibility to the disease.	The incidence of coronary artery disease (CAD).	40 to 69 years at recruitment (baseline).
Ma et al., 2024 [43]	UK Biobank/ UK	Prospective cohort study.	The analytical sample consisted of 452,196 participants from the UK Biobank.	The study examined long-term exposure to: PM2.5, PM10, Nitrogen dioxide (NO2), Nitrogen oxides (NOx)	The primary health outcome was the incidence of stroke, further categorized into ischemic and hemorrhagic stroke.	30 to 73 years at baseline (recruitment).
Liu et al., 2024 [44]	UK Biobank/ UK	Prospective cohort study.	Large sample size (485,288 participants) from the UK Biobank.	Long-term exposure to air pollutants, including: Nitrogen dioxide (NO2), Nitrogen oxides (NOx), Particulate matter with a diameter of 2.5 micrometers or less (PM2.5), Particulate matter with a diameter of 10 micrometers or less (PM10)	The primary health outcome of interest was the incidence of schizophrenia, which was identified through hospital records and self-reported diagnoses in the UK Biobank.	37 to 73 years at the time of recruitment (baseline).
Huang et al., 2024 [45]	Qingdao, China.	Prospective cohort study.	Large sample size of over 312,000 participants.	Long-term exposure to air pollutants, particularly:<br>- Fine particulate matter (PM2.5)<br>- Nitrogen dioxide (NO2)<br>- Nitrogen oxides (NOx)<br>**Genetic Variable:** Polygenic risk score (PRS) for PD	The primary health outcome of interest was Parkinson's disease (PD) diagnosed by neurologists.	Average age of the participants was 57 years old

Wang et al., 2022 [46]	UK Biobank/ UK	Prospective cohort study.	approximately 452,762 participants across the UK. (UK Biobank)	Long-term exposure to air pollution, particularly PM2.5.<br>Genetic Variable: Not directly measured, but genetic susceptibility for COPD was estimated based on the participants' region of residence (a proxy).<br>Lifestyle Variables: Smoking status, alcohol consumption, diet, and physical activity level.	The primary health outcome of interest was chronic obstructive pulmonary disease (COPD).	37 to 73 years.
Rhee et al., 2024 [47]	UK Biobank, which genetically unrelated White British participants without CVD.	Prospective cohort study.	A total of 249 082 participants	Long-term exposure to air pollutants, primarily PM2.5. Other pollutants were likely considered, but PM2.5 was the focus.<br>Genetic Variable: Polygenic risk score (GRS) for CVD, representing an individual's genetic predisposition to the disease.	The primary health outcome was the incidence of cardiovascular diseases (CVD). This included various conditions such as coronary heart disease, stroke, and heart failure.	Aged 40 to 69 Years (2006–2010).
Li et al., 2022 [48]	China	Prospective cohort study.	41,149 participants recruited from the project of Prediction for Atherosclerotic Cardiovascular Disease Risk in China (China-PAR) were included.	Long-term exposure to fine particulate matter (PM2.5).<br>Genetic Variables:<br>Genetic risk scores for coronary artery disease.<br>Residential PM2.5 concentrations.	The primary health outcome investigated was coronary artery disease (CAD), focusing on how long-term exposure to fine particulate matter (PM2.5) and genetic predisposition influence the risk of developing CAD.	40 to 69 years at recruitment.
Chen et al., 2024 [49]	The data primarily comes from the UK Biobank	Prospective cohort study.	The observational analyses involved a large sample size of 453,919 individuals. The genetic analyses focused on individuals of White European descent.	Air Pollution: This was the main exposure of interest. The study examined several air pollutants:<br>Nitrogen oxides (NOx),<br>Nitrogen dioxide (NO2)<br>Particulate matter with a diameter of 2.5 micrometers or less (PM2.5)	The primary health outcome studied was the incidence of ulcerative colitis (UC).	between 40 and 69 years at recruitment.
Wu et al., 2024 [50]	UK Biobank (United Kingdom)	Prospective cohort using data from the UK Biobank.	The study used data from the UK Biobank. The sample size for the observational analyses included over 400,000 participants.<br>The genetic analyses were conducted on a subset of participants of European ancestry.	Primary Exposures:<br>Nitrogen dioxide (NO2), particulate matter ≤2.5 µm (PM2.5)<br>Particulate matter ≤10 µm (PM10)<br>Other Variables:<br>Polygenic risk score (PRS) for psoriasis (measuring genetic predisposition)	The primary health outcome was incident psoriasis, defined as a first diagnosis of psoriasis during the follow-up period in the UK Biobank.	aged between 40 and 69 years at the time of recruitment (between 2006 and 2010).

Zhang et al., 2024 [51]	Beijing, China	Cross-sectional study using data from the UK Bio-Bank.	522 healthy participants living in Beijing from January 2014 to July 2016	Primary Exposure: Particulate matter ≤2.5 µm (PM2.5)<br>Other Variables:<br>Polygenic risk score (PRS) for depression (measuring genetic predisposition)<br>Processing speed (cognitive outcome)<br>Resting-state functional connectivity of the occipitoparietal network and spontaneous activity in the precuneus (neuroimaging measures)<br>Covariates: age, sex, education, smoking status, body mass index, and socioeconomic status	The primary health outcome was processing speed, a measure of cognitive function.	aged between 40 and 69 years at recruitment.
Gao et al., 2023 [52]	UK Biobank (United Kingdom)	Prospective cohort study.	The study included 502,536 participants from the UK Biobank, recruited in 2006–2010.	Researchers estimated participants' long-term exposure to air pollutants:<br>Fine particulate matter (PM2.5)<br>Coarse particulate matter (PM10)<br>Nitrogen oxides (NOx)<br>Nitrogen dioxide (NO2)	Depression & anxiety were assessed using hospital admission records & mental health questionnaires (PHQ-4) at baseline. PHQ-9 (depression & anxiety) & GAD-7 (anxiety) completed during a 7-y follow-up, w/ score cut-offs.	Age of 37–73 years old (baseline survey)
Zhang et al., 2024 [53]	UK (UK Biobank participants)	Prospective cohort study.	Data from the UK Biobank was used. The study included 401,244 participants.	Primary Exposures:<br>Fine particulate matter (PM2.5)<br>Nitrogen dioxide (NO2)<br>Nitrogen oxides (NOx)<br>Joint exposure to these pollutants (analyzed using a weighted quantile sum (WQS) regression)<br>Genetic Susceptibility: APOE ε4 allele (a genetic variant associated with increased dementia risk)	Outcome: Incident dementia	aged between 40 and 69 years at recruitment.

Note: Abbreviations: AAA, Abdominal Aortic Aneurysm; AF, Atrial Fibrillation; CAD, Coronary Artery Disease; CAT, Catalase; COPD, Chronic Obstructive Pulmonary Disease; CVD, Cardiovascular Disease; FT3, Free Triiodothyronine; FT4, Free Thyroxine; MDA, Malondialdehyde; MDD, Major Depressive Disorder; NO2, Nitrogen Dioxide; NOx, Nitrogen Oxides; PM10, Particulate Matter ≤ 10 µm; PM2.5, Particulate Matter ≤ 2.5 µm; PRS, Polygenic Risk Score; SLE, Systemic Lupus Erythematosus; TAA, Total Antioxidant Activity; TPO, Thyroid Peroxidase; TSH, Thyroid-Stimulating Hormone.

Supplementary File S2. Methodological Quality Assessment of the 16 Included Studies Based on Study Design.

1. Methodological Appraisal of Gruzieva et al. (2016) using AMSTAR 2:

- The study was a **meta-analysis based on harmonized individual-level data** from multiple large prospective cohort studies.
- **Eligibility criteria** were clearly defined, and a consistent epigenome-wide association approach (EWAS) was applied across cohorts.
- **Exposure (NO_2) and outcome (DNA methylation)** definitions were standardized, and confounders were controlled for using multivariable regression.
- The study **clearly described the data sources, selection of cohorts, and harmonization methods**.
- Although **a registered protocol was not cited**, the study followed a **transparent and replicable analytic framework**.
- **Risk of bias across cohorts** was minimized by centralized statistical analysis and inclusion of sensitivity checks.
- No evidence of **publication bias** assessment was reported, though this is less applicable in IPD meta-analyses where all data sources are known.

Conclusion:

Based on the AMSTAR 2 framework, *Gruzieva et al. (2016)* demonstrates **high methodological quality** as a meta-analysis of IPD from cohort studies, despite the absence of a formal protocol registration.

2. Methodological Appraisal of Huang et al. (2021)–Lung Cancer Using NOS:

Based on an assessment of the article by Huang et al. (2021), this study is a prospective cohort study utilizing data from the UK Biobank, with a large sample size (over 400,000 participants) and highly comprehensive analyses. The study employed Cox proportional hazards models, controlled for potential confounding variables, conducted sensitivity analyses, and evaluated additive interactions between genetic factors and air pollution exposure.

Assessment using the Newcastle-Ottawa Scale (NOS)

Below is the scoring based on the NOS for cohort studies:

1. Selection (maximum 4 points):

• Representativeness of the exposed cohort: Yes (1 point).
• Selection of the non-exposed cohort: Yes (1 point).
• Ascertainment of exposure: Yes (1 point).
• Demonstration that the outcome of interest was not present at the start: Yes (1 point).

Total: 4/4

2. Comparability (maximum 2 points):

• The study controls for the most important factor (e.g., smoking): Yes.
• The study controls for additional factors (e.g., socioeconomic status, education): Yes.
Total: 2/2

3. Outcome (maximum 3 points):

• Assessment of outcome: Yes (via national cancer registry) (1 point).
• Was follow-up long enough for outcomes to occur: Yes (median follow-up of 7 years) (1 point).
• Adequacy of follow-up: Yes (large number of outcome cases, highly adequate) (1 point).
Total: 3/3
Total NOS Score: 9/9

Therefore, Huang et al. (2021) should be awarded a score of 9 out of 9. This score reflects a very **high methodological quality**.

3. Methodological Appraisal of Ma et al. (2023)–Abdominal Aortic Aneurysm (AAA) Using NOS:

Based on an evaluation of the article by Ma et al. (2023), titled *"Air pollutants, genetic susceptibility, and abdominal aortic aneurysm risk: a prospective study,"* the study employed a **prospective cohort design.** Therefore, the appropriate tool for assessing methodological quality is the **Newcastle-Ottawa Scale (NOS).**

The study used data from the UK Biobank, involving more than 449,000 participants. It applied Cox proportional hazards models with comprehensive adjustment for covariates, including genetic factors, environmental exposures, and sociodemographic characteristics.

Preliminary NOS Assessment:

Based on the information provided in the article:

- **Selection domain:**
  - Representativeness of the exposed cohort √
  - Selection of the non-exposed cohort √
  - Ascertainment of exposure √
  - Demonstration that the outcome was not present at baseline √
- **Comparability domain:**
  - Control for confounding variables (age, sex, lifestyle, etc.) √√
- **Outcome domain:**
  - Assessment of outcome (via hospital record linkage) √
  - Follow-up duration sufficient for outcomes to occur (median follow-up ~ 12.7 years) √

- Adequacy of follow-up (minimal loss to follow-up in UK Biobank) √

Total NOS Score: 9/9

NOS Summary–Ma et al. (2023):

- Selection (4/4):
  √ Representativeness of the exposed cohort.
  √ Selection of the non-exposed cohort.
  √ Exposure ascertainment.
  √ Outcome absent at baseline.
- Comparability (2/2):
  √ Adjusted for key confounders (genetic risk, air pollution, demographics, etc.).
- Outcome (3/3):
  √ Outcome assessment via linked medical records.
  √ Long follow-up (~12.7 years).
  √ Minimal loss to follow-up.

Final NOS Score: 9/9

4. Methodological Appraisal of Li et al. (2023)–Major Depressive Disorder (MDD) Using NOS:

This is a **prospective cohort study**, and is therefore appropriately assessed using the **Newcastle-Ottawa Scale (NOS)**.

Preliminary NOS Assessment:

Based on the information provided in the article, the following is an initial quality appraisal across the three NOS domains:

1. Selection (maximum 4 points)

- Representativeness of the exposed cohort: (UK Biobank, broadly representative population).
- Selection of the non-exposed cohort: (grouped based on exposure levels).
- Ascertainment of exposure: (air pollution assessed via Land Use Regression modeling).
- Demonstration that outcome was not present at baseline: (participants with MDD excluded at baseline).

Score: 4/4

2. Comparability (maximum 2 points)

- Analysis adjusted for key confounders: (e.g., age, sex, education, income).
- Additional stratification by lifestyle factors and polygenic risk scores (PRS).

Score: 2/2

3. Outcome (maximum 3 points)

- Assessment of outcome: (diagnosis based on ICD-10 via hospital and mortality records.
- Follow-up period sufficient: (median follow-up of 9.7 years).
- Adequacy of follow-up: (large sample size, comprehensive coverage).

Score: 3/3

Total NOS Score: 9/9

5. Methodological Appraisal of Fu et al. (2023)–Coronary Artery Disease (CAD) Using NOS:

1. Selection (4/4)

- **Representativeness of the exposed cohort:** The study utilized data from the UK Biobank, which includes a large and broadly representative general population.
- **Selection of the non-exposed cohort:** The control group was drawn from the same population, ensuring comparability in cohort selection.
- **Ascertainment of exposure:** Air pollution exposure was assessed using a validated Land Use Regression (LUR) model.
- **Demonstration that the outcome of interest was not present at the start of the study:** Participants with coronary artery disease (CAD) at baseline were excluded, ensuring all CAD cases were incident.

2. Comparability (2/2)

- **Comparability of cohorts based on design or analysis:** The analysis controlled for a wide range of confounding factors, including age, sex, deprivation index, and lifestyle variables.
- **Control for additional factors:** The study also examined interactions between air pollution and polygenic risk scores (PRS), demonstrating in-depth analysis of genetic susceptibility.

3. Outcome (3/3)

- **Assessment of outcome:** CAD diagnoses were obtained through linkage to hospital records and mortality data, ensuring high accuracy of outcome ascertainment.
- **Was follow-up long enough for outcomes to occur:** The median follow-up duration was 8.8 years, which is sufficient to observe incident CAD events.
- **Adequacy of follow-up:** Loss to follow-up was minimal, with comprehensive data coverage through the UK Biobank.

Total NOS Score: 9/9

Thus, the study by Fu et al. (2023) meets all NOS criteria with a perfect score, indicating **high methodological quality**.

6. Methodological Appraisal of Ma et al. (2024)–Stroke Using NOS:

1. **Selection (4/4)**
   - **Representativeness of the exposed cohort:** The study used data from the UK Biobank, which includes a large and broadly representative general population.
   - **Selection of the non-exposed cohort:** The control group was drawn from the same population, ensuring comparability in cohort selection.
   - **Ascertainment of exposure:** Air pollution exposure was assessed using a validated Land Use Regression (LUR) model.
   - **Demonstration that the outcome of interest was not present at the start of the study:** Participants with a history of stroke at baseline were excluded, ensuring that all stroke cases were incident.
2. **Comparability (2/2)**
   - **Comparability of cohorts based on design or analysis:** The analysis adjusted for a wide range of confounders, including age, sex, deprivation index, and lifestyle factors.
   - **Control for additional factors:** The study also examined interactions between air pollution and polygenic risk scores (PRS), indicating a detailed analysis of genetic susceptibility.
3. **Outcome (3/3)**
   - **Assessment of outcome:** Stroke diagnoses were ascertained through linkage to hospital records and death registries, ensuring high validity.
   - **Was follow-up long enough for outcomes to occur:** The median follow-up was 11.7 years, which is sufficient to observe stroke incidence.
   - **Adequacy of follow-up:** Loss to follow-up was minimal, and the study benefited from the extensive coverage of the UK Biobank.

Total NOS Score: 9/9

Thus, the study by Ma et al. (2024) satisfies all NOS criteria, indicating **high methodological quality**.

7. Methodological Appraisal of Liu et al. (2024)–Schizophrenia Using NOS:

1. **Selection (4/4):**
   - **Representativeness of the exposed cohort:** Utilized data from the UK Biobank, representing a large, diverse general population.
   - **Selection of the non-exposed cohort:** Participants with low air pollution exposure were selected from the same population, ensuring comparability.
   - **Ascertainment of exposure:** Air pollution exposure was assessed using a validated Land Use Regression (LUR) model.
   - **Outcome not present at baseline:** Individuals with a diagnosis of schizophrenia at baseline were excluded, ensuring incident-only cases.
2. **Comparability (2/2):**
   - The analysis adjusted for major confounders, including age, sex, socioeconomic status (SES), and lifestyle factors.

- The study also examined interactions with polygenic risk scores (PRS), offering insights into gene–environment interplay.

3. Outcome (3/3):

- Schizophrenia outcomes were determined using hospital records and official death registry data.
- Follow-up duration exceeded 10 years, sufficient to detect new-onset schizophrenia.
- Loss to follow-up was minimal due to the high-quality linkage in the UK Biobank dataset.

Total NOS Score: 9/9

The study by Liu et al. (2024) meets all NOS criteria and demonstrates **high methodological quality**.

8. Methodological Appraisal of Huang et al. (2024)–Parkinson's Disease Using NOS:

1. Selection (4/4):

- **Representativeness of the exposed cohort:** The study used data from the UK Biobank, a large and diverse general population cohort — representative of the target population.
- **Selection of the non-exposed cohort:** Participants were grouped into quartiles based on air pollution exposure, with all cohorts drawn from the same base population.
- **Ascertainment of exposure:** Exposure to $PM_{2.5}$ and NO_2 was assessed using validated Land Use Regression (LUR) models.
- **Outcome not present at baseline:** Individuals with a diagnosis of Parkinson's disease at baseline were excluded to ensure incident-only cases.

2. Comparability (2/2):

- The models adjusted for a wide range of potential confounders, including age, sex, education, smoking status, and physical activity.
- The study also assessed both additive and multiplicative interactions between air pollution and polygenic risk score (PRS) for Parkinson's disease.

3. Outcome (3/3):

- **Assessment of outcome:** Parkinson's disease was identified through linked hospital records using ICD-10 codes.
- **Follow-up duration:** The average follow-up was 12.2 years, which is sufficient to capture the development of Parkinson's disease.
- **Adequacy of follow-up:** Near-complete follow-up due to the high-quality and systematically linked data from the UK Biobank.

Total NOS Score: 9/9

The methodological quality of Huang et al. (2024) is excellent, meeting all criteria of the Newcastle-Ottawa Scale.

9. Methodological Appraisal of Wang et al. (2022)–COPD Using NOS:

1. Selection (4/4):

- **Representativeness of the exposed cohort:** The study utilized the UK Biobank, a large and diverse population-based cohort — highly representative of the general population.
- **Selection of the non-exposed cohort:** Participants were grouped into exposure quartiles drawn from the same cohort, ensuring valid internal comparisons.
- **Ascertainment of exposure:** Exposure to $PM_{2.5}$ and NO_2 was estimated using validated Land Use Regression (LUR) models.
- **Outcome not present at baseline:** Individuals with a prior diagnosis of COPD were excluded at the start of follow-up to ensure incident-only cases.

2. **Comparability (2/2):**
   - The analysis adjusted for multiple potential confounders, including age, sex, education, smoking status, physical activity, and other lifestyle factors.
   - The study also explored interactions between polygenic risk score (PRS), lifestyle, and air pollution, adding depth to the analysis.
3. **Outcome (3/3):**
   - **Assessment of outcome:** COPD diagnoses were obtained via hospital records using ICD-10 codes.
   - **Follow-up duration:** Median follow-up was 11.9 years, sufficient to observe disease onset.
   - **Adequacy of follow-up:** Near-complete follow-up due to systematic record linkage within the UK Biobank cohort.

Total NOS Score: 9/9

This study demonstrates **high methodological quality**, satisfying all criteria of the Newcastle-Ottawa Scale.

10. Methodological Appraisal of Rhee et al. (2024)–Cardiovascular Disease (CVD) Using NOS:

1. **Selection (4/4):**
   - **Representativeness of the exposed cohort:** The study utilized data from the UK Biobank, a large, population-based cohort representative of the general UK population.
   - **Selection of the non-exposed cohort:** Participants were compared across different exposure levels within the same cohort, ensuring internal validity.
   - **Ascertainment of exposure:** Air pollution exposure ($PM_{2.5}$, PM_{10}, NO_2, NO_x) was estimated using validated Land Use Regression (LUR) models.
   - **Outcome not present at baseline:** Individuals with pre-existing cardiovascular disease were excluded at baseline, ensuring inclusion of incident cases only.
2. **Comparability (2/2):**
   - The model adjusted for key confounding variables such as age, sex, smoking status, body mass index, physical activity, and other lifestyle-related factors.

- The study explicitly examined interactions between polygenic risk scores (PRS) and air pollution exposure.

3. **Outcome (3/3):**
    - **Assessment of outcome:** CVD diagnoses were obtained via national hospital records and coded using ICD classifications.
    - **Follow-up duration:** Median follow-up exceeded 10 years, adequate for assessing cardiovascular disease incidence.
    - **Adequacy of follow-up:** Loss to follow-up was negligible due to comprehensive data linkage within the UK Biobank.

Total NOS Score: 9/9

The study by Rhee et al. (2024) meets all NOS criteria and is considered **methodologically robust**.

11. Methodological Appraisal of Li et al. (2022)–Coronary Artery Disease (CAD) Using NOS:

1. **Selection (4/4):**
    - **Representativeness of the exposed cohort:** Utilized the UK Biobank, a large and broadly representative general population cohort.
    - **Selection of the non-exposed cohort:** Compared individuals across quartiles of air pollution exposure within the same cohort.
    - **Ascertainment of exposure:** Long-term exposure to $PM_{2.5}$ was assessed using validated Land Use Regression (LUR) models.
    - **Outcome not present at baseline:** Participants with existing CAD at baseline were excluded, ensuring analysis of incident cases.
2. **Comparability (2/2):**
    - Adjusted for key confounders, including age, sex, smoking status, education, and lifestyle factors.
    - Polygenic Risk Score (PRS) was included to assess gene–environment interaction.
3. **Outcome (3/3):**
    - **Assessment of outcome:** CAD diagnoses were obtained from hospital records and national death registries using ICD codes.
    - **Follow-up duration:** The average follow-up period was approximately 11 years, sufficient for CAD incidence.
    - **Adequacy of follow-up:** Minimal loss to follow-up due to systematic medical record linkage in the UK Biobank.

Total NOS Score: 9/9

Li et al. (2022) meets all methodological quality criteria with the **highest possible NOS rating.**

12. Methodological Appraisal of Chen et al. (2024)–Ulcerative colitis Using JBI:

Study Design Consideration:

Although the study is described as a prospective cohort, its structure more closely aligns with a **case-control or nested case-control study**, given the retrospective recruitment of ulcerative colitis (UC) patients and matched controls, and the absence of incident outcome data or prospective follow-up.

Chen et al. investigated the association between air pollution exposure and UC susceptibility, emphasizing epigenetic alterations and genetic susceptibility. Given the study's focus on comparing UC cases with healthy controls and the incorporation of epigenetic analyses, the JBI checklist for case-control studies is more suitable than tools designed for cohort studies, such as the Newcastle-Ottawa Scale (NOS).

Assessment Using JBI Case-Control Checklist:

1. **Were the groups comparable other than the presence of disease?**
   Yes. Cases and controls were demographically matched and recruited from the same institution, reducing selection bias.
2. **Were cases and controls matched appropriately?**
   Yes. Matching was done based on age and sex to ensure comparability.
3. **Were the same criteria used for identification of cases and controls?**
   Yes. UC diagnosis was confirmed by ICD codes and histopathology, ensuring consistent case definition.
4. **Was exposure measured in a standard, valid, and reliable way?**
   Yes. Air pollution exposure was estimated using validated Land Use Regression (LUR) models based on residential address.
5. **Was exposure measured similarly for cases and controls?**
   Yes. Exposure estimation methodology was identical for both groups.
6. **Were confounding factors identified?**
   Yes. Important confounders such as age, smoking, and geographic location were recognized.
7. **Were strategies to deal with confounding factors stated?**
   Partially. Some adjustments were made, but multivariable confounder control was limited.
8. **Were outcomes assessed in a standard, valid, and reliable way?**
   Yes. UC diagnosis was confirmed clinically and pathologically.
9. **Was the exposure period long enough to be meaningful?**
   Unclear. The cross-sectional nature limits temporal assessment of exposure–outcome relationships.
10. **Was appropriate statistical analysis used?**
   Yes. Epigenetic analysis and Mendelian randomization approaches enhanced causal inference despite study design limitations.

Rationale for Using JBI Checklist:

Chen et al. (2024) does not employ a full prospective cohort design but rather a **case-control or nested case-control design within a cohort**, focusing on epigenetic modifications and genetic susceptibility. The Newcastle-Ottawa Scale (NOS) is optimized for cohort or case-control studies with clear temporality and follow-up, which this study lacks.

The JBI checklist for case-control studies better captures:

- The quality of matching cases and controls.

- Standardization of exposure and outcome measurement.
- Handling of confounding in case-control contexts.
- Limitations due to lack of longitudinal exposure assessment.

Thus, the JBI tool provides a more tailored, relevant, and nuanced assessment of methodological quality for Chen et al.'s study, reflecting both its strengths and inherent design limitations.

Overall Quality Assessment:

Based on the JBI case-control checklist, Chen et al. (2024) demonstrate a **generally robust methodological approach** with careful matching of cases and controls, valid and reliable exposure and outcome measurements, and appropriate statistical analyses. However, limitations remain due to the partial control for confounding factors and the unclear temporal relationship between exposure and outcome given the cross-sectional design. These factors moderate the certainty of causal inferences but do not substantially undermine the study's internal validity.

13. Methodological Appraisal of Wu et al. (2024)–Psoriasis Using NOS:

1. **Selection (4/4):**
   - **Representativeness of the exposed cohort:** Wu et al. utilized the UK Biobank, which includes a large, demographically diverse population, enhancing generalizability.
   - **Selection of the non-exposed cohort:** Participants were stratified by levels of air pollution and genetic risk within the same source population.
   - **Ascertainment of exposure:** Exposure to pollutants was assessed using validated models from the UK Department for Environment, Food & Rural Affairs (DEFRA). Genetic susceptibility was derived from GWAS data.
   - **Outcome not present at baseline:** Individuals with pre-existing psoriasis at baseline were excluded to ensure assessment of incident cases only.
2. **Comparability (2/2):**
   - The study controlled for major confounding variables such as age, sex, ethnicity, socioeconomic status, smoking, and comorbidities using a directed acyclic graph (DAG)-based approach.
   - Further adjustments were made for genotyping batch effects and population stratification using principal components analysis.
3. **Outcome (3/3):**
   - **Assessment of outcome:** Psoriasis diagnosis was based on a combination of ICD codes, clinical records, and validated self-reported data.
   - **Follow-up duration:** Follow-up was conducted from baseline to diagnosis, death, or end of the study period in 2020-providing sufficient duration.
   - **Adequacy of follow-up:** No substantial loss to follow-up was reported due to the UK Biobank's comprehensive linkage with health records.

Total NOS Score: 9/9

Classification: *High-Quality Study*

14. Methodological Appraisal of Zhang et al. (2024)–Depression on Processing Speed Using NOS for Cross-Sectional Studies:

1. **Selection (4/5):**
   - **Representativeness of the sample:** Participants were healthy adults recruited from local communities in Beijing, providing reasonable representativeness for the target population.
   - **Sample size justification:** No formal sample size calculation or justification was provided.
   - **Non-respondents:** The study did not report information about participant response rates or missing data due to non-participation.
   - **Exposure assessment:** PM2.5 exposure was estimated using robust regional air quality data from 12 government monitoring stations.
   - **Genetic and epigenetic assessment:** Polygenic Risk Scores (PRS) were calculated using validated GWAS summary statistics via PRSice-2, and DNA methylation was profiled with the Illumina EPIC array — both considered valid methods.
2. **Comparability (2/2):**
   - The study controlled for key confounders including age, genetic background, batch effects, and motion artifacts in fMRI scans. These adjustments were clearly reported and statistically accounted for.
3. **Outcome (3/3):**
   - **Outcome validity and reliability:** Outcomes included regional homogeneity (ReHo), resting-state functional connectivity (rsFC), and cognitive performance — all measured using validated and reproducible imaging and behavioral methods.
   - **Statistical analysis:** Advanced methods such as generalized linear modeling (GLM), partial least squares regression (PLSR), permutation testing (10,000 iterations), and false discovery rate (FDR) correction were used — ensuring statistical rigor.

Total NOS Score: 9/10

Classification: *High-Quality Cross-Sectional Study*

Summary:

- Zhang et al. (2024) presents a technically sophisticated cross-sectional study integrating neuroimaging, genetic, epigenetic, and environmental data.
- Despite the lack of sample size justification and non-response reporting, the study applies highly rigorous measurement and analysis strategies.
- The NOS score of **9 out of 10** confirms it as a **methodologically strong study**, suitable for inclusion in evidence syntheses requiring high-quality observational data.

15. Methodological Appraisal of Gao et al. (2023)–Depression and Anxiety Using NOS:

1. **Selection (4/4):**
   - **Representativeness of the exposed cohort:** Gao et al. used the UK Biobank cohort, a large and demographically diverse sample representing the

general UK population, which supports external validity and generalizability.

- **Selection of the non-exposed cohort:** The study included participants without mental health disorders at baseline, enabling comparison of incident cases across varying exposure and genetic risk levels within the same source population.
- **Ascertainment of exposure:** Long-term exposure to air pollutants (e.g., $PM_{2.5}$, NO_2) was estimated using validated land use regression (LUR) models based on geocoded residential data.
- **Outcome not present at baseline:** Individuals with diagnosed depression or anxiety at baseline were excluded, allowing the study to focus solely on new-onset (incident) cases during follow-up.

2. **Comparability (2/2):**
   - **Control for confounding:** The analyses accounted for key confounding variables including age, sex, ethnicity, socioeconomic status, smoking, alcohol consumption, physical activity, and comorbidities.
   - **Additional adjustments:** Genetic predisposition (polygenic risk scores), population stratification (principal components), and genotyping batch effects were also adjusted for in multivariable models.
3. **Outcome (3/3):**
   - **Assessment of outcome:** Incident depression and anxiety were identified through linked electronic health records and validated questionnaires such as PHQ-9 and GAD-7.
   - **Follow-up duration:** The study followed participants for up to 7 years, a sufficient duration for mental health outcomes to manifest.
   - **Adequacy of follow-up:** The UK Biobank's integration with national health registries ensured minimal loss to follow-up, maintaining the cohort's integrity throughout the observation period.

Total NOS Score: 9/9

Classification: High-Quality Study

16. Methodological Appraisal of Zhang et al. (2024)–Dementia Using NOS:

1. **Selection (4/4):**
   - **Representativeness of the exposed cohort:** Zhang et al. utilized data from the UK Biobank, a large and demographically representative cohort of middle-aged and older adults across the UK, enhancing generalizability.
   - **Selection of the non-exposed cohort:** Participants without dementia were drawn from the same UK Biobank population, allowing for appropriate comparison between exposed and unexposed individuals.
   - **Ascertainment of exposure:** Long-term air pollution exposure was estimated using validated datasets from the UK Department for Environment, Food & Rural Affairs (DEFRA), based on geocoded residential history.

- **Outcome not present at baseline:** Participants with pre-existing dementia at baseline were excluded, ensuring only incident cases were included in the analysis.

2. **Comparability (2/2):**
   - **Control for confounding:** The models adjusted for major confounding variables including age, sex, education, BMI, smoking status, alcohol intake, physical activity, socioeconomic status, and other health indicators.
   - **Additional adjustments:** Advanced imputation techniques and multivariable regression were used to handle missing data and account for residual confounding.
3. **Outcome (3/3):**
   - **Assessment of outcome:** Incident dementia was ascertained via linked hospital and death registry data using validated ICD-9 and ICD-10 diagnostic codes.
   - **Follow-up duration:** Participants were followed for up to 16 years (from baseline to 2022), sufficient to observe onset of dementia.
   - **Adequacy of follow-up:** There was no evidence of significant loss to follow-up due to robust record linkage in the UK Biobank cohort.

Total NOS Score: 9/9

Classification: High-Quality Study

Table S3. Summary of gene-environment interactions for environmental exposure and health outcomes.

Author & year	Genetic Variable	Environmental Variable	GxE interaction/Genetic Susceptibility reported	Direction of Interpretation	Health outcome	p-value of Interaction	Effect Size (Interaction/ Main) with (95% CI)	Study Participants	Notes
**Ma et Al., 2024 [40]**	Polygenic Risk Score (PRS) for Abdominal Aortic Aneurysm (AAA)	PM2.5, PM10, NO2, NOx	No, G × E interaction not assessed. Genetic risk categories (low, intermediate, high) significantly associated with AAA risk ($p < 0.001$).	High levels of air pollutants exposure and high genetic risk had a higher risk of developing AAA.	**Abdominal aortic aneurysm**	Not reported	**Interaction HR: Main HR:** PM 2.5, per SDa increase: HR = 1.21 (1.16, 1.27), $p < 0.001$; PM 10, per SD b increase: HR = 1.21 (1.16, 1.27), $p < 0.001$; NO2, per SDc increase: HR = 1.16 (1.11, 1.22), $p < 0.001$; NOx, per SDd increase: HR = 1.10 (1.05, 1.15), $p < 0.001$	UK Biobank participants	PRS constructed from 31 independent SNPs associated with AAA in individuals of European ancestry (MAF > 0.05, $P < 1 \times 10^{-5}$) from two GWASs. Details of PRS construction and SNP information in Supplementary Materials (Text S1, Table S1). Participants categorized into tertiles based on PRS. Significant interactions were reported on an additive scale, but exact p-values were not provided. See Table S8 for further details.

**Rhee et al., 2024 [47]**	**Coronary Artery Disease:** PRS for Coronary Artery Disease. **Myocardial Infarction:** PRS for Myocardial Infarction. **Any Stroke:** PRS for Any Stroke. **Ischemic Stroke:** PRS for Ischemic Stroke. **Heart Failure:** PRS for Heart Failure. **Atrial Fibrillation:** PRS for Atrial Fibrillation.	Data on both PM2.5 and PM10 were collected; however, the main analysis focused on PM2.5.	Because we lack p-interaction values, we will use "Suspected," Potential, or Possible to indicate that an interaction is suggested but not statistically confirmed. A crucial footnote is necessary to explain this.	The effect of PM2.5 exposure appears stronger in individuals with high genetic risk for [CVD Type].	Incident **Cardiovascular disease.**	Not Available (NA) The p-interaction value was not reported explicitly in this study. Interactions were inferred based on differences in Hazard Ratio (HR) between genetic risk groups, but without formal statistical testing for interactions.	Difference in HR at highest exposure level: 1.0. This is *not* a formal interaction effect size, but rather the difference in HRs at the highest exposure level, as a p-interaction value is not available. This provides a rough estimate of the difference in effect between genetic risk groups, but does not account for data variability. Therefore, this value should be interpreted with caution	249,082 participants. Individuals aged 40 to 69 years (2006–2010).	The *p-interaction* was not reported. The difference in HRs at the highest exposure level is presented as a rough estimate of the interaction effect, but statistical significance cannot be determined. PRS calculated using continuous shrinkage method (PRScs) based on Bayesian regression and continuous shrinkage priors (Ge et al.). SNPs coded 0, 1, or 2 based on risk allele count. Posterior effect sizes from GWAS summary statistics and external LD reference panel data were used. Cox proportional hazards models were used to assess the relationship between PM2.5 exposure, genetic risk (PRS), and cardiovascular outcomes.
**Wang et al., 2022 [46]**	Weighted genetic risk score (GRS) for COPD derived from 22 SNPs associated with COPD in a previous GWAS, using UK Biobank data.	PM2.5, PM10, NO2, NOx	Although genetic susceptibility to COPD was considered, no statistically significant interaction between pollutants and genetic risk was found.	**PM2.5:** Although the interaction between PM2.5 and lifestyle was not statistically significant, there is a trend suggesting that the effect of PM2.5 on COPD risk may be slightly greater in individuals with an unfavorable lifestyle. Further research is needed to confirm these findings. **PM10, NOx, NO2:** There is no statistically significant evidence of interaction between these pollutants and lifestyle. herefore, no interpretation of the direction of interaction can be provided.	Incident **Chronic obstructive pulmonary disease (COPD).**	**PM2,5:** p-interaction = 0.101; **PM10:** p-interaction = 0.753; **NO2:** p-interaction = 0.123; **Nox:** p-interaction = 0.258. The p-interaction values are all greater than 0.05, meaning the differences in HRs between the subgroups are not statistically significant.	Effect modification observed, but not statistically significant (p-interaction > 0.05).	452,762 participants from UK Biobank. Aged from 37 to 73 years old.	This article performed statistical analyses of gene-environment interactions. However, results of these interaction analyses are reported as subgroup specific HRs with p-interaction values, but not as interaction HRs from explicit interaction models. The text and table confirms this focus. Based on the data, HR increases with increasing GRS, suggesting that higher genetic susceptibility generally increases the risk of COPD.

**Fu et al., 2023 [42]**	Polygenic Risk Score (PRS) for CAD based on 40 SNPs from a meta-analysis excluding UK Biobank data.	PM2.5, PM10, NO2, NOx	No significant multiplicative interaction (*p* > 0.05). Significant additive interaction.	Increased CAD risk with higher PM2.5; highest risk in high genetic risk & high PM2.5; synergistic effect; subgroups (female, overweight/obese, smokers) more susceptible; effect attenuated after adjusting for race.	Incident **coronary artery disease (CAD).**	**P-interaction:** 0.211; **P-interaction:** 0.715; **P-interaction:** 0.578; **P-interaction:** 0.851	**Interaction effect: PM2.5: Low Genetic Risk:** Low PM2.5: Ref. High PM2.5: 1.06 [95% CI 1.00–1.12]; **Intermediate Risk:** Low PM2.5: 1.25 (95% CI 1.18–1.31). High PM2.5: 1.30 (95% CI 1.23–1.37). **High Genetic Risk:** Low PM2.5: 1.54 (95% CI 1.46–1.62). High PM2.5: 1.56 (95% CI 1.48–1.64). **PM10: Low Genetic Risk:** Low PM10: Ref. High PM10: 1.02 [95% CI 0.96–1.08]; **Intermediate Risk:** High PM10: 1.26 (95% CI 1.18–1.32). High PM10: 1.28 (95% CI 1.21–1.35). **High Genetic Risk:** High PM10: 1.48 (95% CI 1.41–1.56). High PM10: 1.55 (95% CI 1.48–1.63). **NO2: Low Genetic Risk:** Low NO2: Ref. High NO2: 1.05 [95% CI 0.99–1.11]; **Intermediate Risk:** Low NO2: 1.22 (95% CI 1.16–1.28). High NO2: 1.31 (95% CI 1.24–1.38). **High Genetic Risk:** Low NO2: 1.52 (95% CI 1.45–1.59). High NO2: 1.57 (95% CI 1.49–1.65). **NOx: Low Genetic Risk:** Low NOx: Ref. High NOx: 1.03 [95% CI 0.98–1.09]; **Intermediate Risk:** Low NOx: 1.21 (95% CI 1.15–1.27). High NOx: 1.30 (95% CI 1.24–1.37). **High Genetic Risk:** Low NOx: 1.50 (95% CI 1.43–1.57). High NOx: 1.57 (95% CI 1.49–1.65).	UK Biobank participants	RERI > 0: super additive interaction (risk exceeds sum of individual effects); AP: proportion of cases due to interaction. AP > 0: Proportion of cases attributed to interaction (higher AP indicates a larger HRs from Cox models, adjusted for demographics, lifestyle, SES, study center, baseline health, and genetic covariates. (SES = Socioeconomic Status)

**Li et al., 2022 [48]**	PRS for CAD (540 variants).	Long-term PM2.5 exposure.	Yes (both additive and multiplicative).	Highest CAD risk observed in individuals with both high genetic risk and high PM2.5 exposure.	Incident **Coronary artery disease (CAD).**	p-interaction < 0.001.	The abstract provides the relative excess risk due to interaction (RERI): 2.75 (1.32–4.20). This is a specific measure of additive interaction. The HR (95% CI) for multiplicative interaction was 1.19 (1.10–1.28). p for interaction p < 0.001.	A total of 41,149 participants mainly Han Chinese were included in this analysis.	PRS calculated based on 540 variants. PM2.5 exposure categorized into tertiles. Cox proportional hazards regression models with sub-cohort stratum on a calendar year time scale were used to analyze the association between PM2.5, PRS, and CAD. PM2.5 was analyzed as both continuous (per 10 μg/m³ increment) and categorical (tertiles) variable. Three models with increasing confounder adjustment were used. Schoenfeld residual test performed. Exposure-response modeled using restricted cubic splines.
**Zhang et al., 2024 [53]**	* Genetic Risk Score (PRS)* APOE ε4 APOE ε4 genotype (a well-known genetic risk factor for Alzheimer's disease, a common form of dementia)	Joint exposure to multiple air pollutants (PM2.5, PM10, NO2, and NOx).	Yes, interaction analysis conducted. The effect of air pollution on dementia appears to be modified by both a genetic risk score and APOE ε4 genotype, suggesting a synergistic/enhancing effect. However, the p-interaction and interaction effect size (e.g., interaction HR) are not reported in the abstract.	Synergistic/enhancing. The abstract concludes that joint exposure to air pollutants "substantially increases the risk of dementia, especially among individuals with high genetic susceptibility.	Incident **Dementia.**	**p-interaction: Not reported.**	**Interaction HR** (or other effect size for the interaction): The article does not provide a specific HR or other effect size for the interaction itself. It reports HRs for the main effect of the air pollution score (e.g., HR 1.13 for per IQR increase, HR 1.26 for Q4 vs. Q1), but not a separate HR that quantifies the interaction.	Over half a million participants aged 40–69 years in the UK Biobank data recruited in 2006–2010.	* The study used a weighted air pollution score to represent joint exposure to multiple pollutants. * The authors assessed genetic susceptibility using both a PRS and APOE ε4 genotype. * While the article clearly indicates an interaction was investigated, it lacks key statistical information (p-interaction and interaction HR) to fully evaluate its statistical significance and magnitude. Therefore, the conclusion of a synergistic effect is based on the observed pattern rather than a formal statistical test of interaction.

**Gao et al.,2023 [52]**	A polygenic risk score (PRS) approach, aggregating the effects of multiple genetic variants linked to mental health.	PM2.5, PM10,PM coarse, NO2, NOx.	Yes, interaction analysis conducted. The effect of air pollution on mental disorders appears to be modified by the genetic risk score for mental disorders, suggesting a synergistic/enhancing effect. However, the p-interaction and interaction effect size (e.g., interaction HR) are not reported in the abstract.	Suggestive of synergistic/enhancing interaction (effect of air pollution appears stronger in individuals with higher genetic risk).	**Depression and Anxiety*** Prevalent (at baseline) assessed by: o Hospital admission records Mental health questionnaires* Incident (during follow-up) assessed by: o Hospital admission records. Mental health questionnaires	**p-interaction: Not reported.**	The article reports HRs for the main effects of air pollutants but does not report an HR (or any other effect size measure) specifically for the interaction. An interaction HR would quantify how the effect of air pollution changes depending on the genetic risk score. This is essential for understanding the magnitude of the interaction.	398,241 participants from the UK Biobank.	The abstract indicates that a gene-environment interaction was investigated, with findings suggesting that genetic predisposition to mental disorders may enhance the effects of air pollution. However, the abstract does not provide key information necessary for a full evaluation of the interaction: * **Specifics of the PRS:** Details regarding the SNPs included in the polygenic risk score (PRS) or its calculation method are not provided. * **p-interaction:** A p-value for the interaction term is not reported, precluding a definitive assessment of statistical significance. * **Interaction effect size:** No interaction HR (or other measure of effect size for the interaction) is reported, making it impossible to determine the magnitude of the interaction. * **Method of interaction analysis**: The specific statistical method used to test the interaction is not described.

**Gruzieva et al., 2016 [38]**	-	NO2	No GxE interaction.	Effect NO2 exposure on DNA methylation in mitochondria related genes	**Differential DNA methylation** at specific CpG sites in cord blood of newborns.	The article does not report any p-interaction values.	Epigenome-wide significant associations [false discovery rate (FDR) $p < 0.05$] between maternal NO2 exposure during pregnancy and DNA methylation in newborns for 3 CpG sites. The associations they found were statistically significant after correcting for multiple comparisons. However, the abstract does not provide specific effect size measures like beta coefficients, odds ratios, or hazard ratios that would quantify the magnitude of the change in methylation associated with NO2 exposure.	1,508 newborn babies in four European and North American studies	These participants were newborn babies. They were assessed using cord blood DNA methylation. Cord blood is collected at the time of birth, so this sample represents newborns. This is the primary analysis group for the epigenome-wide association study (EWAS). **n = 733 (4 years old)** and **n = 786 (8 years old):** These were subsequent look-up analyses. Prenatal NO2 exposure and DNA methylation (an epigenetic modification) were assessed in newborns; no GxE interaction analyzed. Methylation was analyzed at specific CpG sites.
**Huang et al., 2021 [39]**	**18 SNPs:** Based on 18 single nucleotide polymorphisms (SNPs) reported in the largest lung cancer GWAS of European descent (International Lung Cancer Consortium). **Polygenic Risk** Score (PRS): A polygenic risk score (PRS) was constructed based on these 18 SNPs and categorized into low, intermediate, and high genetic risk based on tertiles of the PRS distribution among non-cases.	PM2.5, PM10, NO2, NOx	Yes, the article states that there are additive interactions between air pollutants and genetic risk.	Effect of PM2.5 on lung cancer risk stronger in individuals with high PRS	**Lung cancer**	NR (not reported)	**PM2.5:** Interm. PRS: RERI 0.36, AP 0.26; High PRS: RERI 0.37, AP 0.21. Positive additive interaction. **PM10:** Interm. PRS: RERI −0.03, AP −0.02; High PRS: RERI 0.11, AP 0.06. No significant additive interaction. **NO2:** Interm. PRS: RERI 0.07, AP 0.05; High PRS: RERI 0.26, AP 0.15. Positive additive interaction in High PRS. **NOx:** Interm. PRS: RERI 0.33, AP 0.26; High PRS: RERI 0.53, AP 0.32. Positive additive interaction.	455,974 UK Biobank participants	PRS is likely calculated based on multiple SNPs; need to verify the details of the article. Analysis may be stratified by lung cancer type. Associations and interactions were assessed using Cox proportional hazards regression models, adjusted for relevant covariates.

**Li et al., 2023 [41]**	A polygenic risk score (PRS) was defined using 17 MDD-associated genetic loci.	PM2.5, PM10, NO2, NOx Annual average concentrations of pollutants were estimated using a Land Use Regression model.	Yes, participants were stratified into groups based on combinations of genetic risk (low, intermediate, high) and environmental exposure (e.g., high/low PM2.5) to assess combined effects	The effect of air pollution on MDD risk tends to be stronger in individuals with high genetic risk (synergistic interaction).	**Major depressive disorder (MDD)**	**PM2.5:** P-interaction = 0.036 (p-value < 0.05); **PM10:** P-interaction = 0.025; (p-value < 0.05); **NO2:** P-interaction = 0.030; (p-value < 0.05); **NOx:** P-interaction = 0.080; (p-value > 0.05)	**Interaction HR: Main HR: PM2.5, per 5-μg/m³** increase: **Model 1**: HR = 1.92 (95% CI: 1.79–2.07), p < 0.001; **Model 2**: HR = 1.16 (95% CI: 1.07–1.26), p < 0.001; **PM10, per 10-μg/m3** increase: **Model 1**: HR = 1.30, (95% CI: 1.20–1.41), p < 0.001; **Model 2**: HR = 1.00, (95% CI: .92–1.09), p = 0.610; **NO2, per 10-μg/m3** increase: **Model 1**: HR = 1.15, (95% CI: 1.13–1.17), p < 0.001; **Model 2**: HR = 1.00, (95% CI: 0.98–1.02), p = 0.695; **NOx, per 20-μg/m3** increase: **Model 1**: HR = 1.15, (95% CI: 1.13–1.17), p < 0.001; **Model 2**: HR = 1.02, (95% CI: 1.02–1.05), p = 0.017	UK Biobank participants	Significant synergistic interaction (p < 0.05). Highlights the importanceof reducing PM2.5 exposure, especially for genetically suscep tible individuals. Significant synergistic interaction (p < 0.05), further reinforces the link air pollution and MDD risk in genetically vulnerable individuals. Significant synergistic interaction (p < 0.05). Suggests a shared biological pathway involving genetic predisposition and NO2 exposure. No significant interaction detected (p > 0.05). Further research is needed to explore the potential role of NOx in MDD development.

**Huang et al., 2024 [45]**	**1. SNPs (Single Nucleotide Polymorphisms):** The specific SNPs used in the study, which in this case are 44 SNPs associated with Parkinson's disease (PD), derived from the PD GWAS. **2. β Coefficients:** The log-odds ratio (β) per allele for each SNP, which represents the risk associated with the SNP for PD, obtained from the relevant GWAS study. **3, Number** of Risk Alleles (SNPi): The count of risk alleles (0, 1, or 2) for each SNP in each individual. **4. Total Number of SNPs (n):** The total number of SNPs used in the analysis, which is 44 in this study.	PM2.5, PM10, NO2, NOx. The annual average air pollution concentration was intricately calculated using an advanced land use regression (LUR) model.	G x E interaction: Not Reported, but genetic susceptibility reported.	HR increases with increasing GRS, indicating that higher genetic susceptibility generally increases the risk of Parkinson's disease.	Incident **Parkinson's disease (PD).**	p-interaction: not available. Figure 1 resents visual evidence of potential interaction; however, the p-value for the interaction term is necessary to determine statistical significance. This value is not shown in the article.	While Figure 1 displays hazard ratios (HRs) for each combination of pollution exposure and genetic risk group, it does not explicitly provide an "interaction HR. To quantify the effect size of the interaction, a formal statistical analysis is needed. The figure suggests a modest effect modification, with the HR increasing more steeply across pollution quartiles in the high genetic risk group compared to the low genetic risk group.	The population-based study involved 312,009 initially PD-free participants with complete genotyping data.	While the authors performed statistical analyses of gene-environment interactions using Cox proportional hazards models (including calculating p-interaction values), Figure 1 focuses on visualizing main effects of genetic risk and stratifying participants by genetic risk level, rather than presenting interaction effects. Consequently, specific results of the interaction analyses (e.g., p-values, interaction HRs) are not reported in the context of Figure 1.

**Zhang et al., 2024 [51]**	Polygenic Risk Score (PRS) of Major Depressive Disorder (MDD).	Fine particulate matter (PM2.5) exposure (average over 6 months).	Yes. The study investigated the interactive effect of air pollution (PM2.5) and genetic risk for MDD (PRS) on processing speed. They found that: * In individuals with high genetic risk for MDD, higher PM2.5 exposure was associated with reduced precuneus connectivity. * Genetic risk for MDD amplified the effect of PM2.5 on DMN connectivity, especially in frontal-parietal and frontal-limbic regions. * In genetically predisposed individuals, higher PM2.5 was linked to increased connectivity between the left angular and cuneus gyri, which in turn was associated with slower processing speed.	The interaction suggests that genetic predisposition to MDD combined with higher PM2.5 exposure has a synergistic effect. It worsens the negative impact of air pollution on brain function (reduced local connectivity in precuneus, increased connectivity in DMN) and processing speed.	**Processing speed.**	The article mentions specific p-values for the interaction effects: * Precuneus local connectivity: PFWE = 0.028. * Default mode network connectivity: PFDR < 0.05.	The article doesn't provide specific effect sizes, but it describes the direction of the interaction (worsening effect with combined exposure and high genetic risk).	497 healthy adult volunteers (48.7% male, mean age 24.5 years) living in Beijing for at least 1 year.	The study investigated the interactive effect of air pollution (PM2.5) and genetic risk for MDD on processing speed and resting state brain function using fMRI and cognitive tests. * The study suggests that air pollution may have a stronger negative impact on brain function and processing speed in individuals with a genetic predisposition to depression. * The study suggests that air pollution may have a stronger negative impact on brain function and processing speed in individuals with a genetic predisposition to depression. * The study also explored the potential role of DNA methylation and gene expression of the SLC30A3 gene in this interaction.

**Wu et al., 2024 [50]**	PRS for Psoriasis.	PM10, PM2.5, NO2, NOx	Yes (multiplicative interaction between PM10 and genetic predisposition).	A multiplicative interaction between PM10 and genetic predisposition (P for interaction = .002)	Incident **Psoriasis**	**PM10: P-interaction (Multiplicative):** 0.002; **P-interaction (Additive):** Not reported. **PM2.5: P-interaction:** 0.105; **P-interaction (Additive):** Not reported. **NO2: P-interaction:** 0.051; **P-interaction (Additive):** Not reported. **PM10: P-interaction:** 0.053; **P-interaction (Additive):** Not reported.	Multiplicative: HR 1.75 (1.25–2.45). The most substantial risk of psoriasis development was observed in participants exposed to elevated air pollution levels combined with high genetic risk.	474 055 individuals with a mean (SD) age of 56.54 (8.09) years.	Time-varying Cox proportional hazards models used. Adjusted for various confounders. Analysis restricted to White European ethnicity.
**Liu et al., 2024 [44]**	PRS for schizophrenia.	PM2.5, PM10, NO2, NOx	There was a significant interaction only for NO2 and NOx exposure, indicating that the effect of exposure on schizophrenia risk depends on the individual's level of genetic susceptibility. Individuals with high genetic risk for schizophrenia are more affected by NO2 and NOx exposure, highlighting the importance of gene-environ ment interactions in schizophrenia risk.	Synergistic (multiplicative), with higher genetic risk amplifies the impact of air pollution exposure (NO2 and NOx) on schizophrenia risk. The adverse effects of air pollution are more pronounced in individuals with greater genetic susceptibility.	Incident **shizophrenia.**	**PM2.5:** p-interaction = 0.48; **PM10:** p-interaction = 0.79; **NO2:** p-interaction = < 0.07; **NOx:** p-interaction = < 0.01. This indicates the genetic risk for schizophrenia does not significantly modify the effect of PM2.5 and PM10 on schizophrenia risk.	The **Effect size** should represent the observed strength of the association between genetic risk, air pollution exposure, and schizophrenia risk. **1. Low genetic risk, T3 HR:** PM2.5: 2.33 (95% CI: 1.93–2.96). PM10: 2.69 (95% CI: 2.09–3.46). NO2: 2.40 (95% CI: 1.37–3.07). NOx: 2.60 (95% CI: 1.94–3.22). **2. High genetic risk, T3 HR:** PM2.5: 6.25 (95% CI: 5.03–7.7). PM10: 7.36 (95% CI: 5.86–9.29). NO2: 6.31 (95% CI: 5.02–7.93). NOx: 6.62 (95% CI: 5.24–8.37). p-value for trends < 0.001 for 4 pollutants.	485,288 participants from UK Biobank.	Multiplicative interactions were assessed using Cox proportional hazards models with a product term for exposure and genetic risk. The reported p-interaction values represent the statistical significance of this interaction term. The pro portional hazards assumption was checked and met. Hazard ratios (HR) and 95% confidence intervals (95% CI) are provided for main effects and, where applicable, for the combined effect of exposure and genetic risk.

**Ma et al., 2024 [43]**	Polygenic Risk Score (PRS) for Stroke, using 71 independent SNPs associated with stroke in European ancestry populations.	PM2.5, PM10, NO2, NOx	While visual inspection of hazard ratios suggested a potential combined effect of air pollution and genetic risk on stroke, no statistically significant multiplicative interaction was found (all p-interaction > 0.05). However, some evidence of additive interaction was observed.	direction of the interaction is synergistic or multiplicative.	Incident **stroke, ischemic stroke, and hemorrhagic stroke**	**PM2.5:** p-interaction = 0.11; **PM10:** p-interaction = 0.75; **NO2:** p-interaction = 0.02; **NOx:** p-interaction = 0.87	Visually stronger exposure effect in high genetic risk group. Graph suggests increased exposure effect with higher genetic risk.	UK Biobank participants	Multiplicative interactions were assessed using Cox proportional hazards models with a product term for exposure and genetic risk. The reported p-interaction values represent the statistical significance of this interaction term. The pro portional hazards assumption was checked and met. Hazard ratios (HR) and 95% confidence intervals (95% CI) are provided for main effects and, where applicable, for the combined effect of exposure and genetic risk.
**Chen et al., 2024 [49]**	For UC results: "UC GRS" (or Genetic Risk Score for UC). For CD results: "CD GRS" (or Genetic Risk Score for CD).	NOx exposure, NO2 exposure, PM2.5 exposure, Combined air pollution score.	Yes (Additive and Multiplicative).	Air pollution is associated with increased UC risk, with effects modified by lifestyle and genetic influences. Epigenetic alterations in CXCR2 and MHC class III region are implicated as potential mechanisms.	Incident **ulcerative colitis (UC).**	P-interaction for multiplicative model = 2.75E−01. P-interaction for additive mo del = 1.23E−03	**Multiplicative: Interaction:** The effect size for the multiplicative interaction is represented by the Interaction HR (95% CI). However, the HRs presented in the "Subgroup analysis" section are not the interac tion HRs directly. They are the HRs for the effect of air pollution within each genetic risk group. **Additive Interaction:** The effect size for additive interaction is given by the RERI: 0.95 (95% CI: 0.34–1.57)	A total of 452,012 (895 cases) and 453,199 (2082 cases) participants were eligible for CD and UC analysis, respectively.	GxE interaction observed, with epigenetic alterations in CXCR2 and MHC class III region implicated as potential mechanisms. Epigenetic Mendelian Randomization (MR) analysis was performed. Cox regression, Schoenfeld residuals test, Epigenetic MR analysis, Co-localization and gene expression analyses; The main focus of the study was on Ulcerative Colitis, with no significant association found for CD.

Note: Abbreviations: OR, Odds Ratio (a measure of association between an exposure and an outcome); CI, Confidence Interval (a range of values that likely contains the true population parameter); RERI, Relative Excess Risk due to Interaction (the proportion of disease among those with both the exposure and the genotype that is attributable to their interaction); AP, Attributable Proportion due to Interaction (the proportion of disease in the population that is attributable to the interaction between the exposure and genotype); SNP, Single Nucleotide Polymorphism (a variation at a single nucleotide that occurs at a specific position in the genome); PRS, Polygenic Risk Score (a score that estimates an individual's risk of a disease based on their genetic variation); PM < sub > 2.5 < /sub > , Particulate Matter with a diameter ≤ 2.5 µm; PM < sub > 10 < /sub > , Particulate Matter with a diameter ≤ 10 µm; NO < sub > 2 < /sub > , Nitrogen Dioxide; NO < sub > x < /sub > , Nitrogen Oxides; GWAS, Genome-Wide Association Study; GxE, Gene-Environment Interaction.

Table S4. Summary of key findings, conclusions, and limitations of included studies.

Author & year	Key Findings	Conclusion	Limitations
Gruzieva et al., 2016 [38]	Epigenome-wide significant associations between maternal NO2 exposure during pregnancy and DNA methylation in newborns for 3 CpG sites in mitochondria related genes (LONP1, HIBADH, SLC25A28). Association with SLC25A28	This study conducted an epigenomewide meta-analysis to identify DNA methylation sites in newborns potentially associated with prenatal exposure to nitrogen dioxide (NO2), a traffic-related air pollutant. They found no statistically significant associations between NO2 exposure and DNA methylation across the entire genome.	This study was limited by potential insufficient statistical power (due to sample size), its focus on cord blood DNA methylation (which may not reflect all relevant changes), incomplete understanding of the link between DNA methylation and health, the exclusive focus on NO2 (other pollutants may have stronger effects), and the assessment being limited to newborns. Despite finding epigenome -wide significant associations at three CpG sites, no genome-wide significant associations were observed.
Huang et al., 2021 [39]	Significant associations between lung cancer risk and PM2.5, PM10, NO2, and NOx. Additive interactions between air pollutants and genetic risk; Highest risk observed in participants with combined high exposure and high genetic risk.	This study provides strong evidence that long-term exposure to air pollutants, particularly PM2.5, NO2, and NOx, significantly increases the risk of lung cancer. It demonstrates a combined effect of genetic predisposition and air pollution, with individuals at high genetic risk and high pollution exposure facing the greatest risk.	This study was limited by the difficulty in isolating individual pollutant effects, the use of a single baseline pollution measurement, the lack of occupational exposure data, simplified smoking data, the lack of oxidative damage biomarkers, and the use of multiple imputation for missing data.
Ma et al., 2024 [40]	Long-term exposure to PM2.5, PM10, NO2, and NOx associated with an increased AAA risk; highest risk in in participants with combined high exposure and high genetic risk.	Long-term exposure to air pollutants, particularly NO2, NOx, PM2.5, and PM10, is associated with an increased risk of abdo minal aortic aneurysm (AAA). This risk is compounded in AAA.	As an observational study, it demonstrates association but cannot prove causation. The findings require confirmation in other populations.
Li et al., 2023 [41]	Long-term exposure to PM2.5, NO2, and NOx) associated with increased Major Depressive Disorder (MDD) risk; interaction observed between air pollution exposure and genetic predisposition (PRS) to MDD.	The study concluded that both genetic susceptibility and lifestyle factors modify the association between long-term air pollution exposure and MDD. Individuals with a higher genetic risk for MDD are more vulnerable to the effects of air pollution.	This study was limited by reliance on self-reported data (potential recall bias), self-reported MDD diagnoses, the predominantly European ancestry of the study population, and potential residual confounding.
Fu et al., 2023 [42]	Exposure to PM2.5, PM10, NO2, and NOx associated with increased CAD risk; higher genetic risk (PRS) increases susceptibility to air pollution's negative effects; additive gene environment interaction observed.	both air pollution exposure and genetic predisposition play a role in CAD development. However, the impact of air pollution is more pronounced for individuals with a higher genetic risk.<br>This highlights the importance of considering both factors for preventing and managing CAD.	As an observational design, it limits causal inference. Unmeasured confounding factors may be present.
Ma et al., 2024 [43]	Long-term exposure to PM2.5, PM10, NO2, and NOx, associated with increased stroke risk; gene-environment Interaction bserved; higher genetic predisposition (PRS) increases susceptibility.	The study concludes that genetic susceptibility modifies the rela tionship between air pollution exposure and stroke risk. People with a higher genetic risk are more vulnerable to the adverse effects of air pollution, especially regarding ischemic stroke.	As an observational design, it limits causal inference. The primarily East Asian study population may limit generalizability. Residual confounding is possible.
Liu et al., 2024 [44]	Long-term exposure to NO2, NOx, PM2.5, and PM10 associated with Increased schizophrenia risk; signficant interaction between genetic susceptibility (PRS) and air	The study concludes that both air pollution exposure and genetic predisposition play a role in the development of	As an observational design, it limits causal inference. The primarily European ancestry of the study

	pollution; highest risk in individuals with combined high risk and high exposure.	schizophrenia. However, the impact of air pollution is more pronounced for individuals with a higher genetic risk. This highlights the importance of considering both factors for risk assessment, prevention, and potentially developing targeted interventions for those at greatest risk.	population limits generalizability. Residual confounding is possible.
Huang et al., 2024 [45]	- No significant association between Air pollution and PD risk in general population; interaction found be tween genetic susceptibility and air pollution; higher genetic riskincreases susceptibility; high genetic risk + exposure to PM2.5, NO2, and NOx associated with increased PD risk.	The study suggests that air pollution might be a risk factor for PD, but its impact is limited to individuals with a preexisting genetic vulnerability. People with a high genetic risk for PD should be more aware of their environmental exposures and consider measures to reduce air pollution intake.	As an observational design, it limits causal inference. Other factors could be influencing PD development. Specific genetic markers associated with PD risk are not specified. The study population was recruited from a single city in China, limiting generalizability.
Wang et al., 2022 [46]	Interaction between air pollution, genetic susceptibility,and lifestyle factors for COPD risk; highest risk observed in individuals with combined high air pollution, high genetic risk, and unhealthy lifestyle.	Air pollution exposure is a risk factor for COPD, but its impact is more pronounced for individuals with a higher genetic predisposition to the disease or those with unhealthy lifestyles. This highlights the importance of considering these combined factors for COPD prevention and risk management.	As an observational design, it limits causal inference. Other factors might influence COPD development. Reliance on self-reported lifestyle data may be prone to bias. The study population was primarily from China, limiting generalizability.
Rhee et al., 2024 [47]	Long-term PM2.5 exposure associated with increased CVD risk; significant interaction between genetic risk (GRS) and air pollution; combined high risk and high exposure resulted in substantially higher CVD risk.	Both genetic predisposition and long-term exposure to air pollution contribute to the development of CVD. The impact of air pollution is amplified in individuals with a higher genetic risk, emphasizing the importance of considering gene-environment interactions in CVD prevention and risk management.	As an observational design, it limits causal inference. The study population consisted primarily of individuals of East Asian descent, limiting generalizability. Air pollution exposure assessment was based on residential address. Genetic risk assessment was based on a polygenic risk score (GRS), which may not fully represent all genetic contributions to to CVD risk.
Li et al., 2022 [48]	Long-term PM2.5 exposure associated with increased CAD risk; high genetic risk for CAD exacerbates PM2.5's adverse effects.	The findings indicate that genetic risk modifies the effect of longterm PM2.5 exposure on coronary artery disease. Individuals with higher genetic susceptibility to CAD are more vulnerable to the detrimental effects of PM2.5. This underscores the importance of considering both environmental and genetic factors in assessing cardiovascular disease risk.	This study was limited by its observational nature (cannot establish causality), potential residual confounding factors, and PM2.5 exposure assessment based on residential addresses.
Chen et al., 2024 [49]	Air pollution increases UC risk by altering DNA methylation, of CXCR2 (involved in immune cell movement) and MHC class III region genes (like AGPAT1). These changes, validated by multiple analyses, affect gene expression in colon tissue and are more pronounced in UC patients' epithelial cells, suggesting a key mechanism linking air pollution to UC development.	The study provides evidence for exposure and the development of ulcerative colitis (UC). This link is mediated, at least in part, by epigenetic alterations, specifically DNA methylation changes, affecting genes like CXCR2 and loci within the MHC class III region. These epigenetic changes influence gene expression in tissue and are more pronounced in UC patients. The findings highlight a potential mechanism by which environmental factors like air pollution can contribute to the pathogenesis of UC.	The observational nature of some analyses limits causal inference. The study population was primarily of White European descent, limiting generalizability. Residual confounding is possible. While Mendelian randomization strengthens causal inference, it relies on certain assumptions. The study focused primarily on UC, with limited analysis of Crohn's disease (CD). The colocalization analysis for cg16689962 could not be performed due to a limited number of mQTLs.

Wu et al., 2024 [50]	Long-term exposure to air pollutants (NO2, PM2.5, PM10) is associated with an increased risk of psoriasis. Genetic susceptibility exacerbates this risk. Mendelian randomization analyses suggest a potential causal role of NO2 and PM2.5 in psoriasis development.	Long-term exposure to air pollution, particularly NO2 and PM2.5, isassociated with an increased risk of psoriasis. Genetic susceptibility to psoriasis interacts with air pollution exposure, exacerbating the risk. This suggests that air pollution may be a trigger for psoriasis in genetically predisposed individuals.	The study population was predominantly White British, limiting generalizability to other ethnicities. Psoriasis diagnosis was based on self-report or hospital records, which may introduce misclassification. Residual confounding factor is possible. MR analyses rely on assumptions (e.g., no pleiotropy). The study did not invesgate the effects of specific types of psoriasis. Air pollution exposure was estimated based on residential address.
Zhang et al., 2024 [51]	Higher PM2.5 exposure was associated with reduced processing speed and precuneus activity (a brain region) in individuals with a high genetic risk for depression.	The study suggests that air pollution may be associated with an increased likelihood of cognitive impairment (specifically reduced processing speed in individuals who are genetically predisposed to depression. This effect may be mediated by alterations in the resting-state function of the occipitoparietal network and the precuneus. These findings highlight the importance of considering gene-environment interactions in understanding the impact of air pollution on brain health.	As a cross-sectional study, it cannot establish causality. It shows associations, but it doesn't prove that air pollution causes reduced processing speed or that the observed brain changes are a direct result of air pollution. The study population is predominantly White British, limiting generalizability. Air pollution exposure was estimated based on residential address, which may not perfectly reflect individual exposure levels. Other potential confounders may not have been fully accounted for.
Gao et al., 2023 [52]	Higher air pollution levels were associated with increased risk of depression and anxiety, both at baseline and during follow-up. This effect may be stronger in individuals with higher genetic risk for depression.	This study suggests a link between long-term air pollution exposure and an increased risk of developing depression and anxiety. Genetic predisposition may play a role in how air pollution affects mental health.	The study relies on estimations of air pollution exposure based on residential address, which may not reflect individual variations.<br>The study design can't definitively prove that air pollution causes depression or anxiety. The focus on genetic predisposition was general, not looking at specific genes. The study population is predominantly White British, limiting generalizability to other ethnicities.
Zhang et al., 2024 [53]	Combined exposure to air pollutants (PM2.5, NO2, NOx) increased dementia risk, especially in individuals carrying the APOE ε4 allele (a genetic risk factor for dementia).	Joint exposure to multiple air pollutants increases the risk of dementia.Genetic susceptibility, particularly carrying the APOE ε4 allele, enhances the detrimental effects of air pollution on dementia risk. This highlights a gene environment interaction.	Exposure to air pollution was estimated based on residential address at baseline, which may not accurately reflect individual exposure over time. Dementia diagnoses were based on administrative data (hospital records and death certificates), which may not capture all cases. The study population is predominantly White British, limiting generalizability to other ethnicities. Residual confounding (influence of other unmeasured factors) is possible.

Note: Abbreviations: AAA, Abdominal Aortic Aneurysm; AF, Atrial Fibrillation; CAD, Coronary Artery Disease; COPD, Chronic Obstructive Pulmonary Disease; CpG sites, Cytosine-phosphate-guanine sites; CVD, Cardiovascular Disease; FT3, Free Triiodothyronine; FT4, Free Thyroxine; GRS, Genetic Risk Score; HIBADH, 3-Hydroxyisobutyrate dehydrogenase; LONP1, Lon peptidase 1, mitochondrial; MDA, Malondialdehyde; MDD, Major Depressive Disorder; NO < sub > 2 < /sub > , Nitrogen Dioxide; NOx, Nitrogen Oxides; PM < sub > 10 < /sub > , Particulate Matter ≤ 10 μm; PM < sub > 2.5 <

/sub > , Particulate Matter ≤ 2.5 μm; PRS, Polygenic Risk Score; mQTLs, methylation Quantitative Trait Loci; SLC25A28, Solute carrier family 25 member 28; SLE, Systemic Lupus Erythematosus; TAA, Total Antioxidant Activity; TSH, Thyroid-Stimulating Hormone; T3, Triiodothyronine; APOE ε4 allele, Apolipoprotein E ε4 allele.

AIMS Press

© 2025 the Author(s), licensee AIMS Press. This is an open access article distributed under the terms of the Creative Commons Attribution License (https://creativecommons.org/licenses/by/4.0)
